# Exploring the training of chinese medical staff oriented to the need for clinical drug information services: from the perspective of drug information patients obtained and need

**DOI:** 10.1186/s12909-023-04680-9

**Published:** 2023-10-06

**Authors:** Rong Huang, Yuankai Huang, Jiayuan Liu, Lei Chen

**Affiliations:** NMPA Key Laboratory for Drug Regulatory Innovation and Evaluation, China Pharmaceutical University, Longmian Avenue 639, Jiangning District, Nanjing, China

**Keywords:** Medical staff, Drug Information Service, Capacity training, Information obtained, Information needs

## Abstract

**Background:**

There are some gaps between the training of drug information service competencies for medical staff and drug information patients need in China.

**Objective:**

To investigate drug information patients obtained and need for further providing directions for the training of drug information service competencies among medical staff in China from patients’ perspectives.

**Methods:**

A face-to-face nationwide survey was conducted using a stratified sampling method. Data were analyzed descriptively using frequencies, percentages and mean. Several subgroup analyses using Chi-square tests were conducted to identify patients’ need for drug information in China.

**Results:**

A total of 1994 questionnaires from medical institutions in China were returned. Most of the drug information obtained by patients came from physicians, and different types of drug information were important to patients. Additionally, patients had different needs for drug information due to age, gender, diagnosis and treatment status, and education level.

**Conclusions:**

The training of medical staff needs to increase the presence of nurses and pharmacists in drug information services, enhance the awareness of “patient-centered” services, and improve the ability to provide information services specific to the characteristics of patients.

**Supplementary Information:**

The online version contains supplementary material available at 10.1186/s12909-023-04680-9.

## Introduction

Drug information service means that physicians, nurse practitioners, physician assistants and other medical staffs provide patients and their families with timely, accurate and comprehensive drug-related information with professional knowledge [1]. The lack of drug information communication between medical staff and patients may lead to treatment failure, increasing risk of disease recurrence, drug-related adverse reactions and additional costs [2].

It is important to provide patients with adequate and appropriate information in the medical process. With the popularization of medical and health knowledge and the improvement of national self-care awareness, patients are more actively involved in the treatment process, and the need for drug information increased meanwhile. However, studies from many countries had found that a considerable number of patients did not get enough drug-related guidance from doctors or pharmacists [3–5] and the majority of patients have unmet drug information needs [3, 6]. The inadequate training of drug information service ability may result in the inability of hospital doctors, pharmacists and pharmacies to meet the public demand for drug information services [7] and even lead to drug safety problems such as excessive self-medication, delayed treatment, drug abuse, and wasted medical resources.

Training of drug information service ability of medical staff is the key to satisfy the unmet drug-related information needs of patients. Unfortunately, the Chinese training model for medical staff focuses on theoretical learning and has not yet completely changed from “drug-centered” to “patient-centered“ [8], resulting in a lack of experience in clinical drug information services and the absence of understanding of the actual needs of clinical patients [9]. The training of medical staff ‘s ability in China is mainly based on undergraduate and master’s degree education, and the drug information service ability is integrated into the academic education system as a part, which is like the United States, Britain, Germany and other countries in the world. In the clinical setting, the Federal Union of German Associations of Pharmacists and British Drug Information Pharmacists Group had developed a series of guidelines on the provision of medication information for pharmacists [10], however, the training mode of clinical pharmacy talents in China remains in the exploratory stage.

Investigating the need of patients for drug information may be one of the main entry points for improving the training program of clinical medical staff in China. Patients wanted more drug information and preferred healthcare providers as a source [11], and there were differences in access to information and the type of drug information needed for patients with different characteristics [12–15]. What’s more, the information provided by medical staff also has emphasis, and some studies have indicated that physicians could explain side effects and pharmacists could provide adequate counseling on certain important issues in providing drug information to patient [16]. However, gaps still exist in the current drug information ability of service providers and the unmet needs of patients, and what competencies medical staff should focus on and what drug information that patients really need remain unclear in China [9].

Therefore, this study aims to investigate the need fulfillment of Chinese patients’ pharmacy information and to distinguish the information preferences of different types of patients, and further providing a reference direction for the training of drug information service capabilities of medical staff.

## Methods

### Questionnaire development

The study focused on various types of specific drug information. To understand the respondents’ sources of obtaining drug information, patients were asked if they had acquired any type of drug information and from which kind of health care provider. To understand the respondents’ need for each type of drug information, we asked the extent of importance of each type of drug information. In order to design each type of drug information covered in the questionnaire, we mainly refer to the literature in English used for instrument development, which provided basic items of patients’ informational needs and medication related problems [17]. Subsequently, the items were translated independently by a native speaker of English who is fluent in Chinese and a Chinese translator and proofed by the expert panel and researchers. To improve the appropriateness and correctness of the questionnaire, the items and options were adjusted according to policies, literature and the advice of experts [13, 17–19]. Based on the literature and the advice of experts [13, 19], all items of the survey questionnaire were divided into five categories (general information, usage information, information on adverse reactions, information involving effects on daily life, and other information) and 27 items(detailed descriptions of the 27 items can be found in the table in the results section). The eventual survey questionnaire was divided into 2 sections.

Section 1 Demographics, including sex, age, diagnosis and treatment status, and level of education.

Sections 2 27 items that contained two questions in each item: [1] whether the respondents had been provided with drug information and their drug information sources (For example, whether time of onset of effectiveness of drug had been provided and who was the information provider. Sources of drug information available to Chinese patients may be doctor/clinical/pharmacist/pharmacy-based pharmacist/nurse/others) [2]. the degree of respondents’ needs for drug information. The options for the first question were as follows: not obtaining this information, doctor, clinical pharmacist, pharmacy pharmacist, nurse, others. The options for the second question were as follows: very unimportant, unimportant, difficult to judge, important, very important. The English versions of the questionnaire was shown in the supplementary material 1.

In May 2019, a pilot test including 20 patients was performed. After the questionnaire was modified based on the results of the pilot test, a pre-test was conducted by convenient sampling to verify the validity and reliability of the questionnaire. The entry criteria for the pilot test and pre-test were the same as those for the actual survey.

The reliability and validity of the “need for drug information” part were tested. The reliability of the questionnaire was tested using the alpha reliability coefficient method, and the validity was tested using the KMO test and the Bartlett’s test of sphericity. The alpha reliability coefficient of the “need for drug information” part was 0.952. The KMO test coefficient was 0.595, and the P value was less than 0.05. The results showed that the reliability and validity of the questionnaire were acceptable.

### Sample design and participant selection

Normally there were few patients in one single primary health care in China, and due to the limited research resources for this study, we surveyed the patients referred from primary health care institutions to secondary or tertiary hospitals to cover as many potential respondents as we could, which was the advice from the experts we consulted when designing the survey. To cover more samples and reflect the overall drug information obtained and needs of patients in China, we used a multistage sampling method to extract samples.

1. Twenty-seven provinces (autonomous regions) and four municipal cities in China were included. Then, all cities were divided into 3 urban groups according to their per capita GDP in 2018 (a total of 93 groups). Next, cities in each province, autonomous region or district in each municipality were evenly divided into 3 groups according to their 2018 per capita GDP, which is associated with the factors influencing patients drug information need, thereby generating 93 groups. Within each group, 1 city or district was selected using the random number method; thus, 93 cities or districts were selected.
2. Based on whether the hospital administrator allowed investigations in the hospital, specific hospitals were selected according to the principle of convenient sampling. At least four secondary hospitals and four tertiary hospitals were selected in each urban group.
3. In each hospital, based on whether two outpatients and one inpatient who were willing to complete the survey could be found, patients were selected by convenience sampling. It is expected that 93*8*3 = 2232 patients will be invited to participate in the study. The data collection assistants entered the hospitals to select patients with the permission of the hospital administrators. If the patients met the inclusion criteria, the data collection assistants introduced the nature, purpose, and content of the survey to them and administered the survey after obtaining written informed consent from the patients. Those included were patients referred from primary hospitals. Reponses from pediatric patients and patients with medical conditions limiting their ability to recall or provide information could be replaced by responses from their family members. Informed consent to participate in the study had been obtained from participants (or their parent or legal guardian) and the ethical approval to conduct the pilot survey, pre-test and main survey was granted by the Ethics Committee of China Pharmaceutical University (Project Number: CPU2019015).

### Data collection

510 data collection assistants who were undergraduate students majoring in pharmacy were recruited in June 2019 to administer the survey. Training was conducted in advance to ensure that all data collection assistants were aware of the research background, purpose, methods and etiquette. The formal survey was conducted in July and August 2019. Using an online survey system, the data collection assistants orally interviewed the patients with each item of the questionnaire and recorded their oral responses, and the survey system then directly converted the data into electronic documents. The data collection assistants were not allowed to provide any view on the questionnaire, but only the requirements for questionnaire filling. 23 postgraduate students were recruited to review the uploaded data and immediately return documents with errors or damaged data for correction through return visits.

The survey was administered after patients underwent diagnosis and treatment to prevent the survey activities from affecting the normal diagnosis and treatment routine of the hospitals and to minimize interruptions of the survey by external factors.

### Data entry and data analysis

The final survey results were automatically entered by the survey system and converted into database files that could be identified by the data analysis software.

Descriptive statistical methods were used to analyze the patients’ drug information needs. The numbers and corresponding percentages for the five broad categories were based on the average of the items below the category.

Several studies have shown that different groups of patients vary in whether they obtain drug information, the types of drug information they obtain and their drug-related needs. To verify whether the above differences exist among Chinese patients with different population characteristics, sex, age, diagnosis and treatment status, and level of education were used as the classification criteria for subgroup analysis. The age of the respondents was categorized according to the new age classification standard of the WHO. Cross-tabulation analysis using the chi-square test was applied to compare the differences among different subgroups. Statistical significance was set as P < 0.05. All the data were analyzed with SPSS 24.0.

We reduced the dimensionality of the data on drug information needs in different subgroups to two dimensions (“important” and “not important”) and reduced the dimensionality of the data on drug information obtained in different subgroups to two dimensions (“obtained” and “not obtained”). For detailed data, see supplementary material 2.

## Results

### Demographics

A total of 1994 valid questionnaires were included, with a response rate of 89.3%.238 questionnaires excluded questionnaires due to incomplete date, data file damaged or unable to be fixed or rebuilt by return visits. The demographic data are shown in Table 1. The mean (SD) age of the respondents was 37.06 (15.53 years). 57.9% of respondents were female. 63.90% of respondents from outpatient clinics, 36.10% from hospitalization.53.4% of respondents have a bachelor’s degree or higher.

**Table 1:** Demographics (n(%))

Demographics	n(%)
Sex	
Male	839(42.10)
Female	1155(57.90)
**Age**	
0–17	59(2.96)
18–65	1731(86.81)
66–79	120(6.02)
80–99	22(1.10)
Missing	62(3.11)
**Diagnosis and treatment status**	
Outpatient	1274(63.90)
Inpatient	720(36.10)
**Level of education**	
Primary school education or below	205(10.30)
Junior high school education	314(15.70)
High school education/secondary school education	411(20.60)
College degree	321(16.10)
Bachelor’s degree	701(35.20)
Master’s degree or above	42(2.10)
**Total**	**1994(100.00)**

### Overall drug information obtainment

In addition to general information and usage information, more than 30% of patients did not acquire other types of information The information obtained most by patients was usage information (94.97%), followed by general information (70.40%). Among the specific drug information, the percentage of obtaining of dosage information was the highest (96.14%), and the percentage of obtaining information on the drug’s impact on one’s sex life was the lowest (31.24%).The drug information obtained by patients mainly comes from doctors. The drug information was obtained slightly more frequently from pharmacy-based pharmacists than from clinical pharmacists. Information from clinical pharmacists, nurses, and others was all lower than 10%. Usage information and other information provided by pharmacy-based pharmacists reached 17.92% and 12.31%, respectively (Table 2).

**Table 2:** Drug information obtainment and sources (%)

Items	Drug Information Obtainment and Sources(N = 1994)					
	Not obtained	Doctor	Clinical pharmacist	Pharmacy-based pharmacist	Nurse	Others
**General information**	**29.60**	**44.70**	**6.54**	**9.04**	**3.12**	**7.00**
(1) Name	6.12	66.00	6.27	12.34	4.51	4.76
(2) Expiration date	29.64	20.66	4.81	16.15	4.41	24.32
(3) Efficacy	10.23	68.00	7.52	7.42	3.11	3.71
(4) Mechanism of action	59.53	22.62	6.37	5.07	1.81	4.61
(5) Time of onset of effectiveness	25.33	53.11	7.12	6.62	4.31	3.51
(6) Judgment of onset of effectiveness	28.59	47.09	6.32	5.72	3.66	8.63
(7) Alternative drugs	52.36	29.54	6.92	7.47	1.30	2.41
(8) Interactions	24.97	50.55	7.02	11.53	1.86	4.06
**Usage information**	**5.03**	**61.65**	**6.60**	**17.92**	**5.92**	**2.88**
(9) Usage methods	4.06	55.62	6.72	22.77	7.37	3.46
(10) Duration of use	7.17	69.11	6.07	10.73	4.41	2.51
(11) Dosage	3.86	60.23	7.02	20.26	5.97	2.66
**Information on adverse reactions**	**36.69**	**43.27**	**6.44**	**6.17**	**2.64**	**4.78**
(12) Side effects	23.87	49.30	6.97	8.83	2.91	8.12
(13) Causes of side effects	37.41	40.77	6.47	7.32	2.71	5.32
(14) Ways to cope with side effects	41.42	41.73	5.82	4.41	2.86	3.76
(15) Ways to cope with forgetting/overuse	47.64	35.31	6.62	4.61	2.76	3.06
(16) Effects on kidney/heart/life	41.98	41.83	5.67	5.42	1.50	3.61
(17) Allergies	27.83	50.70	7.12	6.42	3.11	4.81
**Effects on daily life**	**44.14**	**38.19**	**5.13**	**5.30**	**3.18**	**4.05**
(18) Causing drowsiness	36.51	41.78	5.82	5.92	4.16	5.82
(19) Drinking alcohol	24.82	53.56	5.37	6.67	4.06	5.52
(20) Driving	51.40	32.60	4.61	5.12	2.41	3.86
(21) Reaction speed	55.12	29.24	5.42	4.71	2.46	3.06
(22) Diet	28.23	52.01	5.77	6.32	4.66	3.01
(23) Sex life	68.76	19.96	3.81	3.06	1.35	3.06
**Other information**	**39.99**	**34.57**	**4.21**	**12.31**	**2.56**	**6.36**
(24) Access to more drugs	43.68	32.70	4.71	12.99	2.11	3.81
(25) Reimbursement	33.40	34.90	3.86	14.74	2.91	10.18
(26) Reasons for different prescriptions	48.14	41.78	4.01	3.26	0.95	1.86
(27) Preservation	34.75	28.89	4.26	18.25	4.26	9.58

### Overall drug information needs

Patients generally considered all types of drug information to be important. A total of 93.59% of patients considered usage information important and very important, and 81.95% considered information on adverse reactions important and very important. However, less than 75% of patients considered the remaining information important and very important (Table 3).

**Table 3:** Drug information needs (%)

Items	Drug Information Needs(N = 1994)				
	Very unimportant	Unimportant	Difficult to judge	Important	Very important
**General information**	**1.24**	**9.96**	**14.09**	**46.78**	**27.92**
(1) Name	0.85	8.98	11.63	50.80	27.73
(2) Expiration date	0.40	5.52	8.93	47.94	37.21
(3) Efficacy	0.30	2.51	7.42	53.41	36.36
(4) Mechanism of action	4.36	26.53	23.67	29.74	15.70
(5) Time of onset of effectiveness	0.75	8.02	12.64	53.76	24.82
(6) Judgment of onset of effectiveness	0.50	6.67	13.99	52.16	26.68
(7) Alternative drugs	1.81	17.95	25.28	40.17	14.79
(8) Interactions	0.95	3.51	9.18	46.26	40.09
**Usage information**	**0.20**	**1.81**	**4.41**	**51.81**	**41.78**
(9) Usage methods	0.25	1.96	3.96	51.45	42.38
(10) Duration of use	0.20	2.36	5.22	54.11	38.11
(11) Dosage	0.15	1.10	4.06	49.85	44.83
**Information on adverse reactions**	**0.56**	**4.73**	**12.76**	**46.74**	**35.21**
(12) Side effects	0.40	3.36	8.68	51.50	36.06
(13) Causes of side effects	0.55	5.72	13.84)	47.69	32.20
(14) Ways to cope with side effects	0.30	4.36	12.99	46.69	35.66
(15) Ways to cope with forgetting/overuse	0.80	7.02	17.65	44.73	29.79
(16) Effects on kidney/heart/life	0.60	4.21	13.09	41.62	40.47
(17) Allergies	0.70	3.71	10.33	48.19	37.06
**Effects on daily life**	**4.00**	**15.72**	**18.44**	**39.06**	**22.78**
(18) Causing drowsiness	1.60	17.80	15.55	44.73	20.31
(19) Drinking alcohol	3.11	10.73	9.53	44.48	32.15
(20) Driving	5.62	14.69	17.45	35.71	26.53
(21) Reaction speed	2.51	15.80	23.32	37.26	21.11
(22) Diet	1.20	11.08	14.24	49.80	23.67
(23) Sex life	9.97	24.21	30.58	22.36	12.88
**Other information**	**2.13**	**16.14**	**18.14**	**44.01**	**19.58**
(24) Access to more drugs	3.16	21.21	22.07	37.91	15.65
(25) Reimbursement	1.45	11.48	12.79	49.80	24.47
(26) Reasons for different prescriptions	2.51	19.46	24.07	37.16	16.80
(27) Preservation	1.40	12.39	13.64	51.15	21.41

### Drug information obtainment and needs of patients with different characteristics

#### Responses by age

Compared to patients over 65, patients aged 65 or younger obtained more information about a drug’s name(94.41% VS. 61.27%,P = 0.002), expiration date(71.45% VS. 42.65%, P = 0.01), interactions(76.15% VS. 45.10%, P = 0.003), and side effects(77.37% VS. 44.61%, P < 0.001), as well as whether the drug can cause drowsiness (64.36% VS. 37.25%, P = 0.01)and its impact on driving(49.5% VS. 26.96%,P = 0.045), sex life(32.07% VS. 16.67%,P = 0.045). Patients over 65 received more reimbursement (66.26% VS. 52.94%, P = 0.017) information than their counterparts did. (Table 4)

**Table 4:** Age subgroup analysis of patients’ drug information obtained and needs

Items	Percentage obtained			Percentage considered important		
	Age ≤ 65 (N = 1790)	Age > 65 (N = 204)	p	Age ≤ 65 (N = 1790)	Age > 65 (N = 204)	p
**General information**	**70.96%**	**46.38%**		**74.77%**	**49.51%**	
(1) Name	94.41%	61.27%	0.002^*^	78.88%	51.47%	0.168
(2) Expiration date	71.45%	42.65%	0.010^*^	85.87%	52.94%	0.002^*^
(3) Efficacy	90.17%	59.80%	0.106	89.89%	60.78%	0.333
(4) Mechanism of action	40.56%	27.94%	0.922	44.8%	30.88%	0.919
(5) Time of onset of effectiveness	75.08%	49.51%	0.296	78.77%	52.45%	0.34
(6) Judgment of onset of effectiveness	72.07%	50.00%	0.952	78.88%	54.41%	0.841
(7) Alternative drugs	47.77%	34.80%	0.608	54.3%	39.71%	0.528
(8) Interactions	76.15%	45.10%	0.003^*^	86.76%	53.43%	0.001^*^
**Usage information**	**95.23%**	**64.87%**		**93.65%**	**64.05%**	
(9) Usage methods	96.26%	65.20%	0.126	93.69%	65.69%	0.748
(10) Duration of use	93.18%	63.24%	0.293	92.29%	63.24%	0.537
(11) Dosage	96.26%	66.18%	0.478	94.97%	63.24%	0.035^*^
**Information on adverse reactions**	**63.74%**	**42.16%**		**82.02%**	**54.58%**	
(12) Side effects	77.37%	44.61%	0.000^**^	87.88%	56.37%	0.017^*^
(13) Causes of side effects	63.41%	39.22%	0.093	80.11%	50.98%	0.05
(14) Ways to cope with side effects	58.72%	41.18%	0.918	82.46%	54.90%	0.282
(15) Ways to cope with forgetting/overuse	51.79%	40.69%	0.126	74.13%	51.96%	0.893
(16) Effects on kidney/heart/life	58.21%	40.20%	0.914	82.01%	56.37%	0.76
(17) Allergies	72.96%	47.06%	0.169	85.53%	56.86%	0.214
**Effects on daily life**	**56.45%**	**34.80%**		**62.04%**	**37.09%**	
(18) Causing drowsiness	64.36%	37.25%	0.010^*^	64.86%	42.16%	0.303
(19) Drinking alcohol	75.53%	48.53%	0.123	76.65%	49.51%	0.137
(20) Driving	49.5%	26.96%	0.014^*^	63.24%	30.39%	0.000**
(21) Reaction speed	45.36%	29.90%	0.579	58.49%	35.29%	0.07
(22) Diet	71.9%	49.51%	0.844	73.41%	49.02%	0.44
(23) Sex life	32.07%	16.67%	0.045^*^	35.59%	16.18%	0.003^*^
**Other information**	**60.1%**	**4.53%**		**45.38%**	**46.94%**	
(24) Access to more drugs	56.42%	40.20%	0.76	52.68%	40.69%	0.185
(25) Reimbursement	66.26%	52.94%	0.017^*^	73.63%	55.39%	0.12
(26) Reasons for different prescriptions	51.79%	38.73%	0.377	52.85%	42.65%	0.053
(27) Preservation	65.92%	44.12%	0.539	2.35%	49.02%	0.562

**P < 0.001, *P < 0.05

Compared to patients over 65, patients aged 65 or younger needed more information on a drug’s expiration date (85.87% VS. 52.94%, P = 0.002), interactions (86.76% VS. 53.43%, P = 0.001), dosage (94.97% VS. 63.24%,P = 0.035), side effects (87.88% VS. 56.37%.P = 0.017), and impact on driving(63.24% VS. 30.39%,P < 0.001),sex life (35.59% VS. 16.18%,P = 0.003) (Table 4).

#### Responses by sex

Women obtained more drug information on a drug’s name(94.98% VS. 92.37%, P = 0.017), interactions(77.14% VS. 72.11%, P = 0.010), and dosage(96.88% VS. 95.11%,P = 0.043)than men did, whereas men had more drug information on whether they could drink alcohol while taking medicine(78.31%VS.72.9%, P = 0.006), the drug’s impact on driving(52.09%VS. 46.06%,P = 0.008),reaction speed(47.68%VS. 42.86%,P = 0.033), and access to more drugs( 59.12%VS.54.29%, P = 0.032). (Table 5)

**Table 5:** Gender subgroup analysis of patients’ drug information obtained and needs

Items	Percentage obtained			Percentage considered important		
	Female (N = 1155)	Male (N = 839)	p	Female (N = 1155)	Male (N = 839)	p
**General information**	**70.26%**	**70.60%**		**75.73%**	**73.30%**	
(1) Name	94.98%	92.37%	0.017^*^	79.31%	77.47%	0.325
(2) Expiration date	69.44%	71.63%	0.289	86.75%	82.96%	0.019^*^
(3) Efficacy	90.13%	89.27%	0.533	91.95%	86.77%	0.000^**^
(4) Mechanism of action	38.7%	42.91%	0.059^*^	45.45%	45.41%	0.985
(5) Time of onset of effectiveness	73.68%	76.04%	0.231	79.57%	77.23%	0.21
(6) Judgment of onset of effectiveness	70.74%	72.35%	0.432	79.39%	78.07%	0.475
(7) Alternative drugs	47.27%	48.15%	0.698	55.41%	54.35%	0.638
(8) Interactions	77.14%	72.11%	0.010^*^	87.97%	84.15%	0.014^*^
**Usage information**	**87.46%**	**94.16%**		**94.57%**	**92.21%**	
(9) Usage methods	96.1%	95.71%	0.659	94.55%	92.85%	0.12
(10) Duration of use	93.68%	91.66%	0.084	93.42%	90.58%	0.020^*^
(11) Dosage	96.88%	95.11%	0.043^*^	95.76%	93.21%	0.012^*^
**Information on adverse reactions**	**63.19%**	**63.47%**		**83.51%**	**79.80%**	
(12) Side effects	77.06%	74.85%	0.254	88.66%	86.05%	0.082
(13) Causes of side effects	62.51%	62.69%	0.934	81.65%	77.47%	0.022^*^
(14) Ways to cope with side effects	58.7%	58.40%	0.894	84.85%	78.90%	0.001^*^
(15) Ways to cope with forgetting/overuse	52.73%	51.85%	0.698	76.36%	71.99%	0.027^*^
(16) Effects on kidney/heart/life	56.62%	59.95%	0.137	83.03%	80.81%	0.202
(17) Allergies	71.52%	73.06%	0.446	86.49%	83.55%	0.067
**Effects on daily life**	**54.6%**	**57.59%**		**62.22%**	**61.28%**	
(18) Causing drowsiness	63.29%	63.77%	0.827	66.32%	63.29%	0.161
(19) Drinking alcohol	72.9%	78.31%	0.006^*^	76.1%	77.35%	0.515
(20) Driving	46.06%	52.09%	0.008^*^	61.99%	62.57%	0.791
(21) Reaction speed	42.86%	47.68%	0.033^*^	58.35%	58.40%	0.983
(22) Diet	72.12%	71.28%	0.679	76.02%	69.96%	0.003^*^
(23) Sex life	30.39%	32.42%	0.334	34.55%	36.11%	0.469
**Other information**	**59.2%**	**61.11%**		**64.7%**	**62.07%**	
(24) Access to more drugs	54.29%	59.12%	0.032^*^	54.46%	52.32%	0.345
(25) Reimbursement	65.11%	68.65%	0.098	75.06%	73.18%	0.342
(26) Reasons for different prescriptions	51.26%	52.68%	0.529	54.89%	52.68%	0.328
(27) Preservation	66.15%	64.00%	0.321	74.37%	70.08%	0.034^*^

**P < 0.001, *P < 0.05

Women’s needs for drug information were generally higher than men’s needs, especially information about expiration date(86.75%VS.82.96%,P = 0.019), efficacy(91.95% VS.86.77%,P < 0.001), interactions (87.97% VS.84.15%,P = 0.014), duration of use(93.42% VS.90.58%,P = 0.020), dosage(95.76% VS.93.21%,P = 0.012), causes of side effects(81.65% VS.77.47%,P = 0.022), ways to cope with side effects(84.85% VS.78.90%,P = 0.001), ways to cope with forgetting/overuse(76.36% VS.71.99%,P = 0.027), impact on diet( 76.02% VS.69.96%,P = 0.003)and preservation(74.37% VS.70.08%,P = 0.034) (Table 5).

#### Responses by diagnosis and treatment status

Compared with outpatients, inpatients generally obtained more drug information, especially information about mechanisms of action(43.61% VS.38.70% ,P = 0.032), time of onset of effectiveness(77.78% VS.72.92%,P = 0.017), alternative drugs(51.39% VS.45.53%,P = 0.012), usage methods(97.22% VS.95.21%,P = 0.029), access to more drugs(59.44% VS.54.55%,P = 0.034), reimbursement(74.72% VS.62.01%,P < 0.001), most of the information involving adverse reactions and effects on daily life. (Table 6)

**Table 6:** Diagnostic type subgroup analysis of patients’ drug information obtained and needs

Items	Percentage obtained			Percentage considered important		
	Inpatient (N = 720)	Outpatient (N = 1274)	p	Inpatient (N = 720)	Outpatient (N = 1274)	p
**General information**	**71.42%**	**69.83%**		**75.9%**	**74.03%**	
(1) Name	92.64%	94.58%	0.082	80.69%	77.32%	0.078
(2) Expiration date	67.92%	71.74%	0.072	85.83%	84.77%	0.522
(3) Efficacy	89.31%	90.03%	0.607	90.28%	89.48%	0.573
(4) Mechanism of action	43.61%	38.70%	0.032^*^	48.61%	43.64%	0.032^*^
(5) Time of onset of effectiveness	77.78%	72.92%	0.017^*^	79.44%	78.10%	0.482
(6) Judgment of onset of effectiveness	74.03%	69.94%	0.052	79.72%	78.34%	0.467
(7) Alternative drugs	51.39%	45.53%	0.012^*^	56.81%	53.92%	0.214
(8) Interactions	74.72%	75.20%	0.814	85.83%	86.66%	0.607
**Usage information**	**88.73%**	**94.58%**		**93.15%**	**93.83%**	
(9) Usage methods	97.22%	95.21%	0.029^*^	93.06%	94.27%	0.279
(10) Duration of use	94.31%	91.99%	0.055	91.94%	92.39%	0.723
(11) Dosage	95.42%	96.55%	0.209	94.44%	94.82%	0.72
**Information on adverse reactions**	**67.96%**	**60.68%**		**83.38%**	**81.14%**	
(12) Side effects	78.06%	75.04%	0.129	89.31%	86.58%	0.076
(13) Causes of side effects	66.11%	60.60%	0.015^*^	81.67%	78.89%	0.137
(14) Ways to cope with side effects	63.33%	55.89%	0.001^*^	83.89%	81.48%	0.175
(15) Ways to cope with forgetting/overuse	59.86%	48.12%	0.000^**^	75.28%	74.10%	0.561
(16) Effects on kidney/heart/life	65.42%	53.85%	0.000^**^	84.44%	80.77%	0.040^*^
(17) Allergies	75%	70.57%	0.034^*^	85.69%	85.01%	0.678
**Effects on daily life**	**59.81%**	**53.62%**		**62.01%**	**61.72%**	
(18) Causing drowsiness	66.53%	61.77%	0.034^*^	64.72%	65.23%	0.82
(19) Drinking alcohol	77.22%	74.02%	0.112	75.42%	77.32%	0.336
(20) Driving	51.67%	46.86%	0.039^*^	61.39%	62.72%	0.557
(21) Reaction speed	50.97%	41.44%	0.000^**^	57.78%	58.71%	0.684
(22) Diet	76.67%	69.00%	0.000^**^	76.11%	71.98%	0.045^*^
(23) Sex life	35.83%	28.65%	0.001^*^	36.67%	34.38%	0.304
**Other information**	**63.96%**	**57.77%**		**66.6%**	**61.89%**	
(24) Access to more drugs	59.44%	54.55%	0.034^*^	57.08%	51.57%	0.018^*^
(25) Reimbursement	74.72%	62.01%	0.000^**^	79.44%	71.35%	0.000^**^
(26) Reasons for different prescriptions	54.03%	50.63%	0.144	57.92%	51.73%	0.008^*^
(27) Preservation	67.64%	63.89%	0.092	71.94%	72.92%	0.639

**P < 0.001, *P < 0.05

Compared to outpatients, inpatients needed more drug information about mechanisms of action (48.61% VS.43.64%,P = 0.032), effects on kidney /heart /life(84.44% VS.80.77%,P = 0.040), impact on diet(76.11% VS.71.98%,P = 0.045), access to more drugs(57.08% VS. 51.57%, P = 0.018), reimbursement (79.44% VS.71.35%, P < 0.001), and reasons for different prescriptions (57.92% VS.51.73%, P = 0.008) (Table 6).

#### Responses by level of education

Compared to their counterparts, patients with a bachelor’s degree or above were more inclined to obtain the following types of drug information: name(95.39% VS. 92.15%,P = 0.003), expiration date(73.97% VS.66.24% ,P = 0.001), efficacy(91.82% VS.87.42%,P = 0.001), interactions(76.97% VS.72.80%,P = 0.032), duration of use(93.89% VS.91.61%,P = 0.049), side effects(78.67% VS.73.23%,P = 0.004), causes of side effects(65.13% VS.59.68%,P = 0.012), whether they could drink alcohol while taking medicine(76.97% VS.73.12% ,P = 0.047),and the drug’s impact on driving(50.85% VS.46.02%,P = 0.032). Patients with a lower level of education were more likely to obtain drug information on reimbursement (69.25% VS.64.29%, P = 0.019) than were those with a bachelor’s degree or above. (Table 7)

**Table 7:** Education level subgroup analysis of patients’ drug information obtained and needs

Items	Percentage obtained			Percentage considered important		
	Bachelor’s degree or above (N = 1064)	Bachelor’s degree below (N = 930)	p	Bachelor’s degree or above (N = 1064)	Bachelor’s degree below (N = 930)	p
**General information**	**72.02%**	**68.56%**		**76.5%**	**72.65%**	
(1) Name	95.39%	92.15%	0.003^*^	79.89%	76.99%	0.116
(2) Expiration date	73.97%	66.24%	0.000^**^	87.12%	82.90%	0.008
(3) Efficacy	91.82%	87.42%	0.001^*^	91.54%	87.74%	0.005^*^
(4) Mechanism of action	41.82%	38.92%	0.188	46.8%	43.87%	0.189
(5) Time of onset of effectiveness	76.13%	73.01%	0.11	81.11%	75.70%	0.003^*^
(6) Judgment of onset of effectiveness	72.09%	70.65%	0.477	79.98%	77.53%	0.181
(7) Alternative drugs	47.93%	47.31%	0.782	56.2%	53.55%	0.235
(8) Interactions	76.97%	72.80%	0.032^*^	89.38%	82.90%	0.000^**^
**Usage information**	**87.79%**	**94.05%**		**95.05%**	**91.90%**	
(9) Usage methods	96.52%	95.27%	0.157	94.92%	92.58%	0.030^*^
(10) Duration of use	93.89%	91.61%	0.049^*^	94.17%	90.00%	0.001^*^
(11) Dosage	96.9%	95.27%	0.06	96.05%	93.12%	0.004^*^
**Information on adverse reactions**	**63.85%**	**62.69%**		**83.8%**	**79.82%**	
(12) Side effects	78.67%	73.23%	0.004^*^	90.13%	84.62%	0.000^**^
(13) Causes of side effects	65.13%	59.68%	0.012^*^	82.52%	76.88%	0.002^*^
(14) Ways to cope with side effects	58.46%	58.71%	0.91	83.18%	81.40%	0.299
(15) Ways to cope with forgetting/overuse	51.41%	53.44%	0.365	75.94%	72.90%	0.121
(16) Effects on kidney/heart/life	57.24%	58.92%	0.446	83.55%	80.43%	0.07
(17) Allergies	72.18%	72.15%	0.988	87.5%	82.69%	0.003^*^
**Effects on daily life**	**56.91%**	**54.66%**		**64.57%**	**58.69%**	
(18) Causing drowsiness	64.38%	62.47%	0.378	67.58%	62.15%	0.011^*^
(19) Drinking alcohol	76.97%	73.12%	0.047^*^	79.7%	73.12%	0.001^*^
(20) Driving	50.85%	46.02%	0.032^*^	67.11%	56.67%	0.000^**^
(21) Reaction speed	45.58%	44.09%	0.503	60.53%	55.91%	0.037^*^
(22) Diet	71.52%	72.04%	0.797	74.25%	72.58%	0.4
(23) Sex life	32.14%	30.22%	0.354	38.25%	31.72%	0.002^*^
**Other information**	**59.99%**	**60.03%**		**62.76%**	**64.54%**	
(24) Access to more drugs	56.2%	56.45%	0.911	50.94%	56.56%	0.012^*^
(25) Reimbursement	64.29%	69.25%	0.019^*^	71.05%	77.96%	0.000^**^
(26) Reasons for different prescriptions	53.48%	50.00%	0.121	54.23%	53.66%	0.798
(27) Preservation	65.98%	64.41%	0.463	74.81%	70.00%	0.016^*^

**P < 0.001, *P < 0.05

More educated patients had an especially greater need for information on a drug’s expiration date, efficacy(91.54% VS.87.74%,P = 0.005), time of onset of effectiveness(81.11% VS.75.70%,P = 0.003), interactions(89.38% VS. 82.90%,P < 0.001), usage methods(94.92% VS.92.58%,P = 0.030), duration of use(94.17% VS.90.00%,P = 0.001), dosage(96.05% VS.93.12%,P = 0.004), side effects(90.13% VS.84.62%, P < 0.001), causes of side effects(82.52% VS.76.88%,P = 0.002), allergies(87.5% VS.82.69%,P = 0.003), whether the drug can cause drowsiness(67.58% VS.62.15%,P = 0.011), whether they could drink alcohol while taking medicine(79.7%VS.73.12%,P = 0.001), impact on driving(67.11% VS.56.67%, P < 0.001), reaction speed(60.53% VS.55.91%,P = 0.037),sex life (38.25% VS.31.72%,P = 0.002)and preservation(74.81% VS.70.00%,P = 0.016). Patients with a lower level of education were more concerned about access to more drugs (56.56% VS.50.94%, P = 0.012) and reimbursement (71.05% VS.77.96%, P < 0.001) (Table 7).

## Discussion

To provide a reference direction for training medical staff’s drug information service ability in China from the patient’s perspective, this study investigated the drug information obtained and needs among patients in China and emphatically analyzed the different types of drug information obtained and the need of patients with different characteristics. This was the first nationwide study focusing on this topic in China, which can represent the level of drug information obtained and needs of individuals throughout China. The results revealed that drug information obtained by patients was inadequate, and the age, sex, diagnosis and treatment status and education background were associated with types of drug information needed by patients, which suggested that the training of drug information service competency of medical staff needs to be adjusted accordingly.

The role played by pharmacists and nurses in drug information services needs to be strengthened, and joint physicians form a drug information service network. This survey showed that important sources of information for invested patients were physicians and fewer patients used pharmacists and other sources as drug information sources. Typically, pharmacists are an important subject in providing drug information, but this study showed that pharmacists were not the main information channel for patients, which may be related to the lack of professional pharmacists in China and the under-utilized role of Chinese pharmacists in drug information guidance. According to the statistical bulletin on the development of health care in China in 2021 published by the National Health Commission, the number of professional pharmacists in China was 521,000 in 2021, while the population of China has exceeded 1.4 billion and apparently the number of pharmacists was low [20]. In addition, hospitals are the main medical institution for Chinese patients and prescriptions written by hospitals are basically picked up in the hospital. However, with the development of artificial intelligence, human dispensing of drugs has been partly replaced by machines. Therefore, pharmacists’ drug information guidance role was not fully played. Additionally, social pharmacies, as a complement, are also more likely to sell only medicines without any drug information service. what’s worse, pharmacy pharmacists in social pharmacies are not on duty [21]. The use of drug information appears in the entire process of drug use and requires the collaboration of several drug service providers to achieve, including doctors to correctly diagnose and design drug treatment plans, pharmacists to timely and accurately deploy quality pharmaceuticals, nurses to correctly understand medical advice and issue drugs, and patients to follow medical advice and take medication correctly [22]. Therefore, in the future, physicians, pharmacists, and nurses should improve the linkage and coordination of drug information services, fully adapt to the use of artificial intelligence, supplement the deficiencies of artificial intelligence in the process of drug information provision, and establish an all-round network for drug information provision.

The capacity training of medical staff to provide diversified drug information services should be enhanced. The results of this study showed that the drug information provided to patients focuses on drug use, while some important drug information such as adverse effects and effects on life was not offered, which is thought important besides drug use information. Previous studies in foreign countries have also responded to this problem [17, 18, 23, 24]. The reasons were attributed to, for one thing, the disconnect between basic teaching and clinical use of drug among Chinese medical staff, which may lead to bias in medical staff’s perception of patient actual clinical drug information needs [25]. For another, according to the theoretical teaching accepted by medical staff, too much information on adverse reactions affected patients’ medication adherence, but patients indicated that they need detailed and comprehensive information on adverse reactions [24]. In addition, the lack of investment in the construction of pharmacy information services by medical institutions is also an important reason. Medical staff face a generally heavy burden of clinical medical work and insufficient investment in the work of information services [9]. Some future measures can be used in the stage of drug information service capacity training of medical staff, for example, regular surveys of patient information need and regular operational training to acquire diverse drug information to provide comprehensive drug information.

In the future, it should focus on educating medical staff in a certain aspect of information services according to the different types of patients served, providing patients with appropriate drug information and matching patient needs with drug information services [26]. The survey in this study showed differences in the focus of information needs of patients with different characteristics, which include age, gender, and type of hospitalization. For example, in this study it was reflected that patients under 65 years old were more concerned about the side effects of medications and effects on driving than those over 65 years old; men were more concerned about whether they could drink alcohol during medication use than women; and inpatients were more concerned about information on reimbursement, alternative medications, and adverse effects than outpatients. However, the survey results also revealed the gap between the information obtained by patients and patients’ preferences. For instance, patients under the age of 65, with higher education and inpatients prefer information about the influence of drugs on daily life, but the actual percentage of patients obtaining this drug information was quite low. This may be related to the original “drug-centered” model of drug information service training in China [8], which overemphasized theoretical and experimental research education in the 20th century. Although the transition to “patient-centered” has been greatly improved in the 21st century, the original concept of “drug-centered” still has effect [27]. Data from studies have shown that patient-specific drug information services are associated with reduced hospital mortality [10, 28], and it is important to develop the ability of medical staff to provide specific information. Possible measures include establishing a drug information expert organization that provides comprehensive drug information learning and education for workers at all levels in the pharmaceutical industry, constructing a database platform for patient feedback needs based on big data, and training applied staff who are aligned with actual clinical needs.

This study has a few limitations that could be addressed in future studies. First, according to the new United Nations World Health Organization criteria for age classification this study analyzed 18–65 years (young people) as an age subgroup, which may lead to biased results due to the large age span and the large number of patients accounted for in the interval. Second, this study is based on the patient need perspective to explore the future direction of medical staff training and more perspectives such as medical institutions and government need to be further explored. Third, the factors that influence patients’ medication information needs are complex and varied, and there are some of variables that have yet to be collected for this study. For example, different insurance, disease states, insurance reimbursement policies and diseases have different existing awareness among the patient, need for information etc. These need to be explored further.

## Conclusion

Chinese patients rarely obtain drug information services from healthcare professionals other than clinicians. Age, gender, diagnostic category, and educational background affect the type of information needed by patients, and how to recognize and provide comprehensive pharmacy information services needed by patients is a key concern in the training of the pharmacy information service capacity of medical personnel.

### Electronic supplementary material

Below is the link to the electronic supplementary material.

Supplementary Material 1

## Data Availability

The original contributions presented in the study are included in the article, further inquiries can be directed to the corresponding author.

## References

[1] Xie JJ, Ran XG, Luo XM, Zhang YH, Tian BY (2020). Exploring the application of information services of chinese medicine in hospital pharmacies. Electron J Practical Gynecologic Endocrinol.

[2] Wolf MS, Davis TC, Shrank WH, Neuberger M, Parker RM (2006). A critical review of FDA-approved Medication Guides. Patient Educ Couns.

[3] Maywald U, Schindler C, Krappweis J, Kirch W (2004). First patient-centered drug information service in Germany–a descriptive study. The Annals of Pharmacotherapy.

[4] Krska J, Morecroft CW (2013). Informing patients about medicines–a hospital in-patient survey in England. Patient Educ Couns.

[5] Nader F, Mousavizadeh K, Ghafourifar P (2008). Patient sources for drug information in Iran: a questionnaire-based survey. Pharm World Science: PWS.

[6] Maywald U, Schindler C, Bux Y, Kirch W. [Drug information for patients -- unmet needs, evaluation and influence on the compliance]. Deutsche medizinische Wochenschrift (1946). 2005;130(24):1485-90. 10.1055/s-2005-870843 15942836

[7] Li ZX, Liang AP, Zhang H (2018). An analysis of effective ways to improve pharmacy information service capacity. J Shanxi Coll Staff Med.

[8] Wu M, Wei HP, Wang J (2022). Application of Refined Management of Pharmacy Information Services in improving patient satisfaction. J Chin Med Manage.

[9] Ding JM (2010). On the problems and countermeasures of pharmacy information service in China. Med Inform (Midterm).

[10] ASHP guidelines on surgery and anesthesiology pharmaceutical services. American Society of Health-System Pharmacists (1999). Am J health-system Pharmacy: AJHP : Official J Am Soc Health-System Pharmacists.

[11] Sakai H, Umeda M, Okuyama H, Nakamura S (2020). Differences in perception of breast cancer treatment between patients, physicians, and nurses and unmet information needs in Japan. Supportive care in cancer: Official Journal of the Multinational Association of Supportive Care in Cancer.

[12] Astrom K, Carlsson J, Bates I, Webb DG, Duggan C, Sanghani P (2000). Desire for information about drugs. A multi-method study in general medical inpatients. Pharm World Science: PWS.

[13] Gould O, Buckley P, Doucette D (2013). What patients want: preferences regarding hospital pharmacy services. Can J Hosp Pharm.

[14] Geryk LL, Blalock S, DeVellis RF, Morella K, Carpenter DM (2016). Associations between patient characteristics and the amount of arthritis medication information patients receive. J Health Communication.

[15] Huang L, Fan WD, Yu Y, Liu PY, Yan R, Cheng XN (2017). Investigation of the Habits and understanding Situation and demand of knowledge of Drug Use among patients in our hospital. J China Pharm.

[16] Tarn DM, Paterniti DA, Williams BR, Cipri CS, Wenger NS (2009). Which providers should communicate which critical information about a new medication? Patient, pharmacist, and physician perspectives. J Am Geriatr Soc.

[17] Eibergen L, Janssen MJA, Blom L, Karapinar-Carkit F (2018). Informational needs and recall of in-hospital medication changes of recently discharged patients. Res Social Administrative Pharmacy: RSAP.

[18] Borgsteede SD, Karapinar-Carkit F, Hoffmann E, Zoer J, van den Bemt PM (2011). Information needs about medication according to patients discharged from a general hospital. Patient Educ Couns.

[19] Berhane A, Getahun A, Azanaw A, Hamza S (2013). What patients want to know about their medication? A survey of inpatients and outpatients at Gondar University Hospital. High Educ.

[20] National Healthcare Commission of the People’s Republic of China. Statistical bulletin on the development of health care in China in 2021. National Health and Wellness Commission of the People’s Republic of China. [cited 2023 02–15]. Available from: http://www.gov.cn/xinwen/2022-07/12/content_5700670.htm.

[21] Zhang XY (2022). Where the regulation of licensed pharmacists is heading. China Pharm.

[22] Zhang F (2008). Drug Information and Pharmacy Practice. J Practical Med Technol.

[23] McMahon T, Clark CM, Bailie GR. Who provides patients with drug information? British medical journal (clinical research ed). 1987;294(6568):355–6. 10.1136/bmj.294.6568.355 PMC1245360 3101874

[24] Nair K, Dolovich L, Cassels A, McCormack J, Levine M, Gray J (2002). What patients want to know about their medications. Focus group study of patient and clinician perspectives. Can Family Physician Medecin de Famille Canadien.

[25] Liu SB, Yang H, Yang J, Wang DG (2017). The current situation and reflection of continuing medical education in pharmacy in a military medical university. People’s Military Medicine.

[26] Felkey BG, Fox BI (2014). Getting back to the roots of drug information and looking at the future potential. Hosp Pharm.

[27] Hou XL, Wu Z (2015). Review of the development of higher clinical pharmacy education in China and study of countermeasures. Med Philos (A).

[28] Bond CA, Raehl CL, Franke T (1999). Clinical pharmacy services and hospital mortality rates. Pharmacotherapy.

