# Supplementary material for: Exploring the training of chinese medical staff oriented to the need for clinical drug information services: from the perspective of drug information patients obtained and need
Source: BMC Med Educ. 2023 Oct 6;23:739. doi: 10.1186/s12909-023-04680-9 (PMC10559467; doi:10.1186/s12909-023-04680-9)
Supplement: Supplementary file 1 — Supplementary Material 1 [file 12909_2023_4680_MOESM1_ESM.docx]

**Supplementary Material 1：**

**English vision of the questionnaire**

|  | **1.What is your sex?** | □male □female |
| --- | --- | --- |
|  | **2.How old are you?** | years old |
|  | **3.Are you an outpatient or inpatient?** | □outpatient □inpatient |
|  | **4.What is your level of education?** | □primary school education or below □higher school education/secondary school education □high school education/secondary school education □college degree □bachelor's degree □master's degree or above |
| (abbreviations) | **5. During the visit, did you get the following information? If so, who provided it to you? Do you think this information is important?** | |
| (1) Name | (1) What is the name of the drug | □not obtaining this information □doctor □clinical pharmacist □Pharmacy-based pharmacists □nurse □others |
|  |  | □very unimportant □unimportant □difficult to Judge □important □very important |
| (2) Expiration date | (2) What is the expiration date of the drug | □not obtaining this information □doctor □clinical pharmacist □Pharmacy-based pharmacists □nurse □others |
|  |  | □very unimportant □unimportant □difficult to Judge □important □very important |
| (3) Efficacy | (3) What the drug is for | □not obtaining this information □doctor □clinical pharmacist □Pharmacy-based pharmacists □nurse □others |
|  |  | □very unimportant □unimportant □difficult to Judge □important □very important |
| (4) Mechanism of action | (4) How the drug works | □not obtaining this information □doctor □clinical pharmacist □Pharmacy-based pharmacists □nurse □others |
|  |  | □very unimportant □unimportant □difficult to Judge □important □very important |
| (5) Time of onset of effectiveness | (5) How long does it takes before the drug works | □not obtaining this information □doctor □clinical pharmacist □Pharmacy-based pharmacists □nurse □others |
|  |  | □very unimportant □unimportant □difficult to Judge □important □very important |
| (6) Judgment of onset of effectiveness | (6) How to judge if the drug works | □not obtaining this information □doctor □clinical pharmacist □Pharmacy-based pharmacists □nurse □others |
|  |  | □very unimportant □unimportant □difficult to Judge □important □very important |
| (7) Alternative drugs | (7) Whether, and if so what the alternatives are for the drug | □not obtaining this information □doctor □clinical pharmacist □Pharmacy-based pharmacists □nurse □others |
|  |  | □very unimportant □unimportant □difficult to Judge □important □very important |
| (8) Interactions | (8) Whether there are any interactions with other drugs | □not obtaining this information □doctor □clinical pharmacist □Pharmacy-based pharmacists □nurse □others |
|  |  | □very unimportant □unimportant □difficult to Judge □important □very important |
| (9) Usage methods | (9) How to use the drug | □not obtaining this information □doctor □clinical pharmacist □Pharmacy-based pharmacists □nurse □others |
|  |  | □very unimportant □unimportant □difficult to Judge □important □very important |
| (10) Duration of use | (10) How long the drug should be taken | □not obtaining this information □doctor □clinical pharmacist □Pharmacy-based pharmacists □nurse □others |
|  |  | □very unimportant □unimportant □difficult to Judge □important □very important |
| (11) Dosage | (11) How much dose the drug is taken | □not obtaining this information □doctor □clinical pharmacist □Pharmacy-based pharmacists □nurse □others |
|  |  | □very unimportant □unimportant □difficult to Judge □important □very important |
| (12) Side effects | (12) What are the side effects of the drug | □not obtaining this information □doctor □clinical pharmacist □Pharmacy-based pharmacists □nurse □others |
|  |  | □very unimportant □unimportant □difficult to Judge □important □very important |
| (13) Causes of side effects | (13）What will cause the side effects of the drug | □not obtaining this information □doctor □clinical pharmacist □Pharmacy-based pharmacists □nurse □others |
|  |  | □very unimportant □unimportant □difficult to Judge □important □very important |
| (14) Ways to cope with side effects | (14) What to do if you have side effects | □not obtaining this information □doctor □clinical pharmacist □Pharmacy-based pharmacists □nurse □others |
|  |  | □very unimportant □unimportant □difficult to Judge □important □very important |
| (15) Ways to cope with forgetting/overuse | (15) What to do when you forgot to take the drug or when you took too much | □not obtaining this information □doctor □clinical pharmacist □Pharmacy-based pharmacists □nurse □others |
|  |  | □very unimportant □unimportant □difficult to Judge □important □very important |
| (16) Effects on kidney/heart/life | (16) What are the effects on your kidney, heart, and life | □not obtaining this information □doctor □clinical pharmacist □Pharmacy-based pharmacists □nurse □others |
|  |  | □very unimportant □unimportant □difficult to Judge □important □very important |
| (17) Allergies | (17) What are the allergies that the drug can cause | □not obtaining this information □doctor □clinical pharmacist □Pharmacy-based pharmacists □nurse □others |
|  |  | □very unimportant □unimportant □difficult to Judge □important □very important |
| (18) Causing drowsy | (18) Whether the drug can make you feel drowsy | □not obtaining this information □doctor □clinical pharmacist □Pharmacy-based pharmacists □nurse □others |
|  |  | □very unimportant □unimportant □difficult to Judge □important □very important |
| (19) Drinking alcohol | (19) Whether you can drink alcohol whilst taking the drug | □not obtaining this information □doctor □clinical pharmacist □Pharmacy-based pharmacists □nurse □others |
|  |  | □very unimportant □unimportant □difficult to Judge □important □very important |
| (20) Driving | (20) What are the impacts on driving when taking the drug | □not obtaining this information □doctor □clinical pharmacist □Pharmacy-based pharmacists □nurse □others |
|  |  | □very unimportant □unimportant □difficult to Judge □important □very important |
| (21) Reaction ability | (21) What are the impacts on your reaction ability when taking the drug | □not obtaining this information □doctor □clinical pharmacist □Pharmacy-based pharmacists □nurse □others |
|  |  | □very unimportant □unimportant □difficult to Judge □important □very important |
| (22) Diet | (22) What are the impacts on your diet when taking the drug | □not obtaining this information □doctor □clinical pharmacist □Pharmacy-based pharmacists □nurse □others |
|  |  | □very unimportant □unimportant □difficult to Judge □important □very important |
| (23) Sex life | (23) Whether the drug will affect your sex life | □not obtaining this information □doctor □clinical pharmacist □Pharmacy-based pharmacists □nurse □others |
|  |  | □very unimportant □unimportant □difficult to Judge □important □very important |
| (24) Access to more drugs | (24) How to get more drugs | □not obtaining this information □doctor □clinical pharmacist □Pharmacy-based pharmacists □nurse □others |
|  |  | □very unimportant □unimportant □difficult to Judge □important □very important |
| (25) Reimbursement | (25) Whether the drug is reimbursed | □not obtaining this information □doctor □clinical pharmacist □Pharmacy-based pharmacists □nurse □others |
|  |  | □very unimportant □unimportant □difficult to Judge □important □very important |
| (26) Different prescriptions reasons | (26) What are the reasons for different prescriptions when suffering from same illness | □not obtaining this information □doctor □clinical pharmacist □Pharmacy-based pharmacists □nurse □others |
|  |  | □very unimportant □unimportant □difficult to Judge □important □very important |
| (27) Preservation | (27) How to preserve the drug | □not obtaining this information □doctor □clinical pharmacist □Pharmacy-based pharmacists □nurse □others |
|  |  | □very unimportant □unimportant □difficult to Judge □important □very important |

| **Supplementary Material 2: Detailed data of subgroup** | | | | | | | | | | | | | | | | | | | | | | | | |
| --- | --- | --- | --- | --- | --- | --- | --- | --- | --- | --- | --- | --- | --- | --- | --- | --- | --- | --- | --- | --- | --- | --- | --- | --- |
| **Table 1 Drug Information Obtainment of Patients by Age (n(%))** | | | | | | | | | | | | | | | | | | | | | | | | |
|  | **0-17(N=59)** |  |  |  |  |  | **18-65(N=1731)** | |  |  |  |  | **66-79(N=120)** |  |  |  |  |  | **80-99(N=22)** | |  |  |  |  |
|  | **Not Obtained** | **Doctor** | **Clinical Pharmacist** | **Pharmacy- based pharmacist** | **Nurse** | **Others** | **Not Obtained** | **Doctor** | **Clinical Pharmacist** | **Pharmacy- based pharmacist** | **Nurse** | **Others** | **Not Obtained** | **Doctor** | **Clinical Pharmacist** | **Pharmacy- based pharmacist** | **Nurse** | **Others** | **Not Obtained** | **Doctor** | **Clinical Pharmacist** | **Pharmacy-based pharmacist** | **Nurse** | **Others** |
| **General information** |  |  |  |  |  |  |  |  |  |  |  |  |  |  |  |  |  |  |  |  |  |  |  |  |
| (1) What is the name of the drug | 7(11.86) | 39(66.10) | 2(3.39) | 6(10.17) | 3(5.08) | 2(3.39) | 93(5.37) | 1139(65.80) | 113(6.53) | 227(13.11) | 72(4.16) | 87(5.03) | 15(12.50) | 79(65.83) | 5(4.17) | 6(5.00) | 11(9.17) | 4(3.33) | 2(9.09) | 16(72.73) | 1(4.55) | 1(4.55) | 2(9.09) | 0(0.00) |
| (2) What is the expiration date of the drug | 18(30.51) | 11(18.64) | 1(1.69) | 6(10.17) | 4(6.78) | 19(32.20) | 493(28.48) | 354(20.45) | 83(4.79) | 295(17.04) | 73(4.22) | 433(25.01) | 43(35.83) | 32(26.67) | 9(7.50) | 13(10.83) | 5(4.17) | 18(15.00) | 12(54.55) | 4(18.18) | 0(0.00) | 1(4.55) | 2(9.09) | 3(13.64) |
| (3) What the drug is for | 5(8.47) | 39(66.10) | 1(1.69) | 5(8.47) | 3(5.08) | 6(10.17) | 171(9.88) | 1183(68.34) | 136(7.86) | 135(7.80) | 46(2.66) | 60(3.47) | 18(15.00) | 76(63.33) | 6(5.00) | 8(6.67) | 7(5.83) | 5(4.17) | 2(9.09) | 18(81.82) | 1(4.55) | 0(0.00) | 1(4.55) | 0(0.00) |
| (4) How the drug works | 36(61.02) | 14(23.73) | 3(5.08) | 1(1.69) | 1(1.69) | 4(6.78) | 1028(59.39) | 385(22.24) | 110(6.35) | 95(5.49) | 31(1.79) | 82(4.74) | 71(59.17) | 30(25.00) | 9(7.50) | 4(3.33) | 4(3.33) | 2(1.67) | 14(63.64) | 6(27.27) | 1(4.55) | 1(4.55) | 0(0.00) | 0(0.00) |
| (5) How long does it takes before the drug works | 22(37.29) | 25(42.37) | 3(5.08) | 4(6.78) | 3(5.08) | 2(3.39) | 424(24.49) | 929(53.67) | 125(7.22) | 120(6.93) | 74(4.27) | 59(3.41) | 33(27.50) | 65(54.17) | 8(6.67) | 8(6.67) | 3(2.50) | 3(2.50) | 8(36.36) | 11(50.00) | 0(0.00) | 0(0.00) | 3(13.64) | 0(0.00) |
| (6) How to judge if the drug works | 22(37.29) | 22(37.29) | 4(6.78) | 3(5.08) | 3(5.08) | 5(8.47) | 478(27.61) | 821(47.43) | 108(6.24) | 105(6.07) | 65(3.76) | 154(8.90) | 32(26.67) | 61(50.83) | 11(9.17) | 6(5.00) | 3(2.50) | 7(5.83) | 8(36.36) | 12(54.55) | 1(4.55) | 0(0.00) | 1(4.55) | 0(0.00) |
| (7) Whether, and if so what the alternatives are for the drug | 33(55.93) | 11(18.64) | 3(5.08) | 9(15.25) | 1(1.69) | 2(3.39) | 902(52.11) | 513(29.64) | 123(7.11) | 130(7.51) | 21(1.21) | 42(2.43) | 60(50.00) | 42(35.00) | 8(6.67) | 7(5.83) | 3(2.50) | 0(0.00) | 11(50.00) | 9(40.91) | 0(0.00) | 1(4.55) | 0(0.00) | 1(4.55) |
| (8) Whether there are any interactions with other drugs | 24(40.68) | 19(32.20) | 1(1.69) | 7(11.86) | 1(1.69) | 7(11.86) | 403(23.28) | 891(51.47) | 126(7.28) | 214(12.36) | 30(1.73) | 67(3.87) | 39(32.50) | 61(50.83) | 7(5.83) | 5(4.17) | 5(4.17) | 3(2.50) | 11(50.00) | 10(45.45) | 1(4.55) | 0(0.00) | 0(0.00) | 0(0.00) |
| **Usage Information** |  |  |  |  |  |  |  |  |  |  |  |  |  |  |  |  |  |  |  |  |  |  |  |  |
| (9) How to use the drug | 3(5.08) | 34(57.63) | 2(3.39) | 11(18.64) | 4(6.78) | 5(8.47) | 64(3.70) | 939(54.25) | 125(7.22) | 421(24.32) | 120(6.93) | 62(3.58) | 7(5.83) | 78(65.00) | 3(2.50) | 14(11.67) | 17(14.17) | 1(0.83) | 2(9.09) | 16(72.73) | 0(0.00) | 3(13.64) | 1(4.55) | 0(0.00) |
| (10) How long the drug should be taken | 11(18.64) | 35(59.32) | 0(0.00) | 7(11.86) | 3(5.08) | 3(5.08) | 111(6.41) | 1200(69.32) | 111(6.41) | 198(11.44) | 71(4.10) | 40(2.31) | 9(7.50) | 86(71.67) | 6(5.00) | 6(5.00) | 11(9.17) | 2(1.67) | 4(18.18) | 14(63.64) | 0(0.00) | 2(9.09) | 1(4.55) | 1(4.55) |
| (11) How much dose the drug is taken | 4(6.78) | 36(61.02) | 1(1.69) | 11(18.64) | 4(6.78) | 3(5.08) | 63(3.64) | 1023(59.10) | 131(7.57) | 373(21.55) | 98(5.66) | 43(2.48) | 6(5.00) | 79(65.83) | 4(3.33) | 12(10.00) | 15(12.50) | 4(3.33) | 1(4.55) | 18(81.82) | 0(0.00) | 2(9.09) | 0(0.00) | 1(4.55) |
| **Information on adverse reactions** |  |  |  |  |  |  |  |  |  |  |  |  |  |  |  |  |  |  |  |  |  |  |  |  |
| (12) What are the side effects of the drug | 22(37.29) | 21(35.59) | 2(3.39) | 5(8.47) | 1(1.69) | 8(13.56) | 383(22.13) | 866(50.03) | 120(6.93) | 165(9.53) | 51(2.95) | 146(8.43) | 43(35.83) | 59(49.17) | 9(7.50) | 3(2.50) | 4(3.33) | 2(1.67) | 8(36.36) | 10(45.45) | 2(9.09) | 0(0.00) | 1(4.55) | 1(4.55) |
| (13）What will cause the side effects of the drug | 27(45.76) | 16(27.12) | 1(1.69) | 8(13.56) | 3(5.08) | 4(6.78) | 628(36.28) | 720(41.59) | 115(6.64) | 130(7.51) | 47(2.72) | 91(5.26) | 52(43.33) | 47(39.17) | 8(6.67) | 3(2.50) | 3(2.50) | 7(5.83) | 10(45.45) | 9(40.91) | 0(0.00) | 2(9.09) | 0(0.00) | 1(4.55) |
| (14) What to do if you have side effects | 32(54.24) | 19(32.20) | 2(3.39) | 3(5.08) | 1(1.69) | 2(3.39) | 707(40.84) | 724(41.83) | 99(5.72) | 80(4.62) | 54(3.12) | 67(3.87) | 47(39.17) | 54(45.00) | 9(7.50) | 4(3.33) | 1(0.83) | 5(4.17) | 11(50.00) | 10(45.45) | 1(4.55) | 0(0.00) | 0(0.00) | 0(0.00) |
| (15) What to do when you forgot to take the drug or when you took too much | 32(54.24) | 16(27.12) | 4(6.78) | 3(5.08) | 1(1.69) | 3(5.08) | 831(48.01) | 610(35.24) | 115(6.64) | 85(4.91) | 42(2.43) | 48(2.77) | 48(40.00) | 43(35.83) | 10(8.33) | 4(3.33) | 9(7.50) | 6(5.00) | 11(50.00) | 11(50.00) | 0(0.00) | 0(0.00) | 0(0.00) | 0(0.00) |
| (16) What are the effects on your kidney, heart, and life | 30(50.85) | 18(30.51) | 2(3.39) | 4(6.78) | 1(1.69) | 4(6.78) | 718(41.48) | 720(41.59) | 102(5.89) | 101(5.83) | 27(1.56) | 63(3.64) | 48(40.00) | 58(48.33) | 7(5.83) | 3(2.50) | 2(1.67) | 2(1.67) | 12(54.55) | 10(45.45) | 0(0.00) | 0(0.00) | 0(0.00) | 0(0.00) |
| (17) What are the allergies that the drug can cause | 25(42.37) | 23(38.98) | 4(6.78) | 3(5.08) | 1(1.69) | 3(5.08) | 459(26.52) | 890(51.42) | 126(7.28) | 115(6.64) | 56(3.24) | 85(4.91) | 40(33.33) | 61(50.83) | 8(6.67) | 6(5.00) | 2(1.67) | 3(2.50) | 6(27.27) | 13(59.09) | 0(0.00) | 1(4.55) | 2(9.09) | 0(0.00) |
| **Effects on Daily Life** |  |  |  |  |  |  |  |  |  |  |  |  |  |  |  |  |  |  |  |  |  |  |  |  |
| (18) Whether the drug can make you feel drowsy | 25(42.37) | 18(30.51) | 2(3.39) | 4(6.78) | 3(5.08) | 7(11.86) | 613(35.41) | 733(42.35) | 104(6.01) | 109(6.30) | 74(4.27) | 98(5.66) | 54(45.00) | 47(39.17) | 7(5.83) | 2(1.67) | 5(4.17) | 5(4.17) | 12(54.55) | 8(36.36) | 0(0.00) | 1(4.55) | 0(0.00) | 1(4.55) |
| (19) Whether you can drink alcohol whilst taking the drug | 23(38.98) | 23(38.98) | 2(3.39) | 2(3.39) | 2(3.39) | 7(11.86) | 415(23.97) | 933(53.90) | 96(5.55) | 126(7.28) | 70(4.04) | 91(5.26) | 39(32.50) | 63(52.50) | 6(5.00) | 2(1.67) | 5(4.17) | 5(4.17) | 4(18.18) | 13(59.09) | 1(4.55) | 1(4.55) | 2(9.09) | 1(4.55) |
| (20) What are the impacts on driving when taking the  drug | 31(52.54) | 18(30.51) | 1(1.69) | 4(6.78) | 0(0.00) | 5(8.47) | 873(50.43) | 574(33.16) | 81(4.68) | 93(5.37) | 45(2.60) | 65(3.76) | 74(61.67) | 34(28.33) | 6(5.00) | 4(3.33) | 1(0.83) | 1(0.83) | 13(59.09) | 6(27.27) | 0(0.00) | 1(4.55) | 1(4.55) | 1(4.55) |
| (21) What are the impacts on your reaction ability  when taking the drug | 34(57.63) | 12(20.34) | 1(1.69) | 5(8.47) | 2(3.39) | 5(8.47) | 944(54.53) | 510(29.46) | 95(5.49) | 84(4.85) | 44(2.54) | 54(3.12) | 66(55.00) | 40(33.33) | 8(6.67) | 2(1.67) | 3(2.50) | 1(0.83) | 15(68.18) | 6(27.27) | 0(0.00) | 1(4.55) | 0(0.00) | 0(0.00) |
| (22) What are the impacts on your diet when taking  the drug | 15(25.42) | 31(52.54) | 2(3.39) | 5(8.47) | 3(5.08) | 3(5.08) | 488(28.19) | 901(52.05) | 98(5.66) | 117(6.76) | 80(4.62) | 47(2.72) | 34(28.33) | 66(55.00) | 9(7.50) | 2(1.67) | 5(4.17) | 4(3.33) | 7(31.82) | 10(45.45) | 0(0.00) | 0(0.00) | 3(13.64) | 2(9.09) |
| (23) Whether the drug will affect your sex life | 44(74.58) | 7(11.86) | 1(1.69) | 3(5.08) | 1(1.69) | 3(5.08) | 1172(67.71) | 364(21.03) | 66(3.81) | 57(3.29) | 25(1.44) | 47(2.72) | 93(77.50) | 14(11.67) | 6(5.00) | 0(0.00) | 0(0.00) | 7(5.83) | 15(68.18) | 4(18.18) | 1(4.55) | 0(0.00) | 1(4.55) | 1(4.55) |
| **Other information** |  |  |  |  |  |  |  |  |  |  |  |  |  |  |  |  |  |  |  |  |  |  |  |  |
| (24) How to get more drugs | 30(50.85) | 13(22.03) | 1(1.69) | 12(20.34) | 0(0.00) | 3(5.08) | 750(43.33) | 567(32.76) | 78(4.51) | 231(13.34) | 40(2.31) | 65(3.76) | 53(44.17) | 42(35.00) | 8(6.67) | 13(10.83) | 1(0.83) | 3(2.50) | 7(31.82) | 11(50.00) | 1(4.55) | 0(0.00) | 0(0.00) | 3(13.64) |
| (25) Whether the drug is reimbursed | 21(35.59) | 18(30.51) | 1(1.69) | 12(20.34) | 1(1.69) | 6(10.17) | 583(33.68) | 585(33.80) | 69(3.99) | 263(15.19) | 53(3.06) | 178(10.28) | 28(23.33) | 66(55.00) | 5(4.17) | 10(8.33) | 1(0.83) | 10(8.33) | 6(27.27) | 9(40.91) | 0(0.00) | 2(9.09) | 0(0.00) | 5(22.73) |
| (26) What are the reasons for different prescriptions when suffering from same illness | 34(57.63) | 18(30.51) | 3(5.08) | 2(3.39) | 0(0.00) | 2(3.39) | 829(47.89) | 725(41.88) | 71(4.10) | 58(3.35) | 17(0.98) | 31(1.79) | 54(45.00) | 56(46.67) | 3(2.50) | 4(3.33) | 2(1.67) | 1(0.83) | 9(40.91) | 13(59.09) | 0(0.00) | 0(0.00) | 0(0.00) | 0(0.00) |
| (27) How to preserve the drug | 23(38.98) | 13(22.03) | 1(1.69) | 13(22.03) | 1(1.69) | 8(13.56) | 587(33.91) | 500(28.89) | 72(4.16) | 330(19.06) | 74(4.27) | 168(9.71) | 42(35.00) | 46(38.33) | 5(4.17) | 14(11.67) | 6(5.00) | 7(5.83) | 10(45.45) | 5(22.73) | 0(0.00) | 3(13.64) | 2(9.09) | 2(9.09) |

| **Table 2 Drug Information Needs of Patients by Age (n(%))** | | | | | | | | | | | | | | | | | | | | |
| --- | --- | --- | --- | --- | --- | --- | --- | --- | --- | --- | --- | --- | --- | --- | --- | --- | --- | --- | --- | --- |
|  | **0-17(N=59)** |  |  |  |  | **18-65(N=1731)** | |  |  |  | **66-79(N=120)** | |  |  |  | **80-99(N=22)** | |  |  |  |
|  | **Very Unimportant** | **Unimportant** | **Difficult to Judge** | **Important** | **Very Important** | **Very Unimportant** | **Unimportant** | **Difficult to Judge** | **Important** | **Very Important** | **Very Unimportant** | **Unimportant** | **Difficult to Judge** | **Important** | **Very Important** | **Very Unimportant** | **Unimportant** | **Difficult to Judge** | **Important** | **Very Important** |
| **General information** |  |  |  |  |  |  |  |  |  |  |  |  |  |  |  |  |  |  |  |  |
| (1) What is the name of the drug | 1(1.69) | 4(6.78) | 6(10.17) | 28(47.46) | 20(33.90) | 12(0.69) | 160(9.24) | 195(11.27) | 885(51.13) | 479(27.67) | 4(3.33) | 8(6.67) | 20(16.67) | 63(52.50) | 25(20.83) | 0(0.00) | 2(9.09) | 3(13.64) | 12(54.55) | 5(22.73) |
| (2) What is the expiration date of the drug | 0(0.00) | 2(3.39) | 11(18.64) | 22(37.29) | 24(40.68) | 8(0.46) | 94(5.43) | 138(7.97) | 847(48.93) | 644(37.20) | 0(0.00) | 9(7.50) | 19(15.83) | 54(45.00) | 38(31.67) | 0(0.00) | 2(9.09) | 4(18.18) | 8(36.36) | 8(36.36) |
| (3) What the drug is for | 0(0.00) | 1(1.69) | 6(10.17) | 33(55.93) | 19(32.20) | 6(0.35) | 41(2.37) | 127(7.34) | 920(53.15) | 637(36.80) | 0(0.00) | 5(4.17) | 12(10.00) | 63(52.50) | 40(33.33) | 0(0.00) | 0(0.00) | 1(4.55) | 15(68.18) | 6(27.27) |
| (4) How the drug works | 4(6.78) | 13(22.03) | 19(32.20) | 15(25.42) | 8(13.56) | 73(4.22) | 472(27.27) | 407(23.51) | 511(29.52) | 268(15.48) | 7(5.83) | 26(21.67) | 34(28.33) | 35(29.17) | 18(15.00) | 1(4.55) | 5(22.73) | 6(27.27) | 9(40.91) | 1(4.55) |
| (5) How long does it takes before the drug works | 0(0.00) | 7(11.86) | 13(22.03) | 25(42.37) | 14(23.73) | 14(0.81) | 139(8.03) | 207(11.96) | 947(54.71) | 424(24.49) | 1(0.83) | 10(8.33) | 17(14.17) | 60(50.00) | 32(26.67) | 0(0.00) | 1(4.55) | 6(27.27) | 11(50.00) | 4(18.18) |
| (6) How to judge if the drug works | 1(1.69) | 5(8.47) | 11(18.64) | 28(47.46) | 14(23.73) | 9(0.52) | 113(6.53) | 239(13.81) | 912(52.69) | 458(26.46) | 0(0.00) | 10(8.33) | 18(15.00) | 59(49.17) | 33(27.50) | 0(0.00) | 1(4.55) | 2(9.09) | 14(63.64) | 5(22.73) |
| (7) Whether, and if so what the alternatives are for the drug | 3(5.08) | 10(16.95) | 19(32.20) | 19(32.20) | 8(13.56) | 31(1.79) | 318(18.37) | 437(25.25) | 693(40.03) | 252(14.56) | 1(0.83) | 20(16.67) | 33(27.50) | 52(43.33) | 14(11.67) | 0(0.00) | 2(9.09) | 5(22.73) | 10(45.45) | 5(22.73) |
| (8) Whether there are any interactions with other drugs | 3(5.08) | 2(3.39) | 11(18.64) | 24(40.68) | 19(32.20) | 14(0.81) | 56(3.24) | 151(8.72) | 809(46.74) | 701(40.50) | 1(0.83) | 11(9.17) | 16(13.33) | 48(40.00) | 44(36.67) | 0(0.00) | 1(4.55) | 4(18.18) | 9(40.91) | 8(36.36) |
| **Usage Information** |  |  |  |  |  |  |  |  |  |  |  |  |  |  |  |  |  |  |  |  |
| (9) How to use the drug | 0(0.00) | 2(3.39) | 1(1.69) | 29(49.15) | 27(45.76) | 4(0.23) | 35(2.02) | 71(4.10) | 888(51.30) | 733(42.35) | 1(0.83) | 1(0.83) | 5(4.17) | 62(51.67) | 51(42.50) | 0(0.00) | 0(0.00) | 1(4.55) | 15(68.18) | 6(27.27) |
| (10) How long the drug should be taken | 0(0.00) | 2(3.39) | 6(10.17) | 29(49.15) | 22(37.29) | 3(0.17) | 41(2.37) | 86(4.97) | 942(54.42) | 659(38.07) | 1(0.83) | 2(1.67) | 7(5.83) | 63(52.50) | 47(39.17) | 0(0.00) | 1(4.55) | 2(9.09) | 12(54.55) | 7(31.82) |
| (11) How much dose the drug is taken | 0(0.00) | 1(1.69) | 3(5.08) | 34(57.63) | 21(35.59) | 3(0.17) | 16(0.92) | 67(3.87) | 858(49.57) | 787(45.47) | 0(0.00) | 3(2.50) | 9(7.50) | 61(50.83) | 47(39.17) | 0(0.00) | 1(4.55) | 0(0.00) | 14(63.64) | 7(31.82) |
| **Information on adverse reactions** |  |  |  |  |  |  |  |  |  |  |  |  |  |  |  |  |  |  |  |  |
| (12) What are the side effects of the drug | 0(0.00) | 8(13.56) | 8(13.56) | 25(42.37) | 18(30.51) | 7(0.40) | 49(2.83) | 145(8.38) | 900(51.99) | 630(36.40) | 1(0.83) | 9(7.50) | 15(12.50) | 59(49.17) | 36(30.00) | 0(0.00) | 0(0.00) | 2(9.09) | 13(59.09) | 7(31.82) |
| (13）What will cause the side effects of the drug | 1(1.69) | 8(13.56) | 11(18.64) | 22(37.29) | 17(28.81) | 10(0.58) | 93(5.37) | 233(13.46) | 833(48.12) | 562(32.47) | 0(0.00) | 10(8.33) | 23(19.17) | 52(43.33) | 35(29.17) | 0(0.00) | 1(4.55) | 4(18.18) | 13(59.09) | 4(18.18) |
| (14) What to do if you have side effects | 0(0.00) | 8(13.56) | 10(16.95) | 21(35.59) | 20(33.90) | 5(0.29) | 67(3.87) | 224(12.94) | 827(47.78) | 608(35.12) | 0(0.00) | 10(8.33) | 15(12.50) | 45(37.50) | 50(41.67) | 0(0.00) | 1(4.55) | 4(18.18) | 12(54.55) | 5(22.73) |
| (15) What to do when you forgot to take the drug or when you took too much | 2(3.39) | 6(10.17) | 11(18.64) | 21(35.59) | 19(32.20) | 12(0.69) | 118(6.82) | 314(18.14) | 773(44.66) | 514(29.69) | 2(1.67) | 9(7.50) | 19(15.83) | 60(50.00) | 30(25.00) | 0(0.00) | 2(9.09) | 4(18.18) | 11(50.00) | 5(22.73) |
| (16) What are the effects on your kidney, heart, and life | 1(1.69) | 3(5.08) | 10(16.95) | 25(42.37) | 20(33.90) | 9(0.52) | 72(4.16) | 227(13.11) | 717(41.42) | 706(40.79) | 2(1.67) | 6(5.00) | 15(12.50) | 51(42.50) | 46(38.33) | 0(0.00) | 1(4.55) | 3(13.64) | 11(50.00) | 7(31.82) |
| (17) What are the allergies that the drug can cause | 0(0.00) | 3(5.08) | 11(18.64) | 24(40.68) | 21(35.59) | 13(0.75) | 64(3.70) | 168(9.71) | 841(48.58) | 645(37.26) | 1(0.83) | 6(5.00) | 16(13.33) | 52(43.33) | 45(37.50) | 0(0.00) | 0(0.00) | 3(13.64) | 14(63.64) | 5(22.73) |
| **Effects on Daily Life** |  |  |  |  |  |  |  |  |  |  |  |  |  |  |  |  |  |  |  |  |
| (18) Whether the drug can make you feel drowsy | 0(0.00) | 17(28.81) | 11(18.64) | 23(38.98) | 8(13.56) | 30(1.73) | 302(17.45) | 269(15.54) | 781(45.12) | 349(20.16) | 2(1.67) | 24(20.00) | 21(17.50) | 46(38.33) | 27(22.50) | 0(0.00) | 5(22.73) | 4(18.18) | 10(45.45) | 3(13.64) |
| (19) Whether you can drink alcohol whilst taking the drug | 5(8.47) | 8(13.56) | 10(16.95) | 21(35.59) | 15(25.42) | 48(2.77) | 191(11.03) | 156(9.01) | 776(44.83) | 560(32.35) | 7(5.83) | 14(11.67) | 14(11.67) | 48(40.00) | 37(30.83) | 2(9.09) | 0(0.00) | 4(18.18) | 10(45.45) | 6(27.27) |
| (20) What are the impacts on driving when taking the  drug | 7(11.86) | 9(15.25) | 14(23.73) | 19(32.20) | 10(16.95) | 87(5.03) | 248(14.33) | 293(16.93) | 633(36.57) | 470(27.15) | 15(12.50) | 29(24.17) | 26(21.67) | 23(19.17) | 27(22.50) | 2(9.09) | 4(18.18) | 4(18.18) | 9(40.91) | 3(13.64) |
| (21) What are the impacts on your reaction ability  when taking the drug | 3(5.08) | 10(16.95) | 11(18.64) | 24(40.68) | 11(18.64) | 38(2.20) | 272(15.71) | 409(23.63) | 645(37.26) | 367(21.20) | 7(5.83) | 23(19.17) | 26(21.67) | 40(33.33) | 24(20.00) | 1(4.55) | 7(31.82) | 6(27.27) | 6(27.27) | 2(9.09) |
| (22) What are the impacts on your diet when taking  the drug | 0(0.00) | 2(3.39) | 10(16.95) | 33(55.93) | 14(23.73) | 20(1.16) | 201(11.61) | 243(14.04) | 865(49.97) | 402(23.22) | 2(1.67) | 13(10.83) | 18(15.00) | 54(45.00) | 33(27.50) | 1(4.55) | 1(4.55) | 7(31.82) | 11(50.00) | 2(9.09) |
| (23) Whether the drug will affect your sex life | 12(20.34) | 13(22.03) | 21(35.59) | 9(15.25) | 4(6.78) | 154(8.90) | 424(24.49) | 529(30.56) | 398(22.99) | 226(13.06) | 24(20.00) | 33(27.50) | 35(29.17) | 15(12.50) | 13(10.83) | 5(22.73) | 5(22.73) | 7(31.82) | 4(18.18) | 1(4.55) |
| **Other information** |  |  |  |  |  |  |  |  |  |  |  |  |  |  |  |  |  |  |  |  |
| (24) How to get more drugs | 4(6.78) | 11(18.64) | 14(23.73) | 23(38.98) | 7(11.86) | 51(2.95) | 384(22.18) | 383(22.13) | 647(37.38) | 266(15.37) | 6(5.00) | 18(15.00) | 27(22.50) | 48(40.00) | 21(17.50) | 1(4.55) | 1(4.55) | 6(27.27) | 11(50.00) | 3(13.64) |
| (25) Whether the drug is reimbursed | 2(3.39) | 6(10.17) | 8(13.56) | 28(47.46) | 15(25.42) | 22(1.27) | 210(12.13) | 224(12.94) | 867(50.09) | 408(23.57) | 3(2.50) | 8(6.67) | 13(10.83) | 59(49.17) | 37(30.83) | 0(0.00) | 3(13.64) | 2(9.09) | 10(45.45) | 7(31.82) |
| (26) What are the reasons for different prescriptions when suffering from same illness | 5(8.47) | 14(23.73) | 12(20.34) | 20(33.90) | 8(13.56) | 42(2.43) | 341(19.70) | 430(24.84) | 627(36.22) | 291(16.81) | 2(1.67) | 23(19.17) | 22(18.33) | 53(44.17) | 20(16.67) | 0(0.00) | 4(18.18) | 4(18.18) | 12(54.55) | 2(9.09) |
| (27) How to preserve the drug | 0(0.00) | 16(27.12) | 7(11.86) | 23(38.98) | 13(22.03) | 23(1.33) | 205(11.84) | 238(13.75) | 903(52.17) | 362(20.91) | 4(3.33) | 15(12.50) | 14(11.67) | 56(46.67) | 31(25.83) | 1(4.55) | 6(27.27) | 2(9.09) | 10(45.45) | 3(13.64) |

| **Table 3 Drug Information Obtainment of Patients by Sex (n(%))** | | | | | | | | | | | | |
| --- | --- | --- | --- | --- | --- | --- | --- | --- | --- | --- | --- | --- |
|  | **Male(N=839)** |  |  |  |  |  | **Female(N=1155)** | |  |  |  |  |
|  | **Not Obtained** | **Doctor** | **Clinical Pharmacist** | **Pharmacy- based**  **pharmacist** | **Nurse** | **Others** | **Not Obtained** | **Doctor** | **Clinical Pharmacist** | **Pharmacy- based**  **pharmacist** | **Nurse** | **Others** |
| **General information** |  |  |  |  |  |  |  |  |  |  |  |  |
| (1) What is the name of the drug | 64(7.63) | 527(62.81) | 62(7.39) | 100(11.92) | 42(5.01) | 44(5.24) | 58(5.02) | 789(68.31) | 63(5.45) | 146(12.64) | 48(4.16) | 51(4.42) |
| (2) What is the expiration date of the drug | 238(28.37) | 185(22.05) | 43(5.13) | 145(17.28) | 46(5.48) | 182(21.69) | 353(30.56) | 227(19.65) | 53(4.59) | 177(15.32) | 42(3.64) | 303(26.23) |
| (3) What the drug is for | 90(10.73) | 562(66.98) | 71(8.46) | 66(7.87) | 26(3.10) | 24(2.86) | 114(9.87) | 794(68.74) | 79(6.84) | 82(7.10) | 36(3.12) | 50(4.33) |
| (4) How the drug works | 479(57.09) | 202(24.08) | 60(7.15) | 48(5.72) | 20(2.38) | 30(3.58) | 708(61.30) | 249(21.56) | 67(5.80) | 53(4.59) | 16(1.39) | 62(5.37) |
| (5) How long does it takes before the drug works | 201(23.96) | 436(51.97) | 70(8.34) | 62(7.39) | 47(5.60) | 23(2.74) | 304(26.32) | 623(53.94) | 72(6.23) | 70(6.06) | 39(3.38) | 47(4.07) |
| (6) How to judge if the drug works | 232(27.65) | 380(45.29) | 62(7.39) | 54(6.44) | 35(4.17) | 76(9.06) | 338(29.26) | 559(48.40) | 64(5.54) | 60(5.19) | 38(3.29) | 96(8.31) |
| (7) Whether, and if so what the alternatives are for the drug | 435(51.85) | 243(28.96) | 64(7.63) | 67(7.99) | 13(1.55) | 17(2.03) | 609(52.73) | 346(29.96) | 74(6.41) | 82(7.10) | 13(1.13) | 31(2.68) |
| (8) Whether there are any interactions with other drugs | 234(27.89) | 398(47.44) | 64(7.63) | 98(11.68) | 16(1.91) | 29(3.46) | 264(22.86) | 610(52.81) | 76(6.58) | 132(11.43) | 21(1.82) | 52(4.50) |
| **Usage Information** |  |  |  |  |  |  |  |  |  |  |  |  |
| (9) How to use the drug | 36(4.29) | 451(53.75) | 63(7.51) | 197(23.48) | 64(7.63) | 28(3.34) | 45(3.90) | 658(56.97) | 71(6.15) | 257(22.25) | 83(7.19) | 41(3.55) |
| (10) How long the drug should be taken | 70(8.34) | 570(67.94) | 50(5.96) | 93(11.08) | 41(4.89) | 15(1.79) | 73(6.32) | 808(69.96) | 71(6.15) | 121(10.48) | 47(4.07) | 35(3.03) |
| (11) How much dose the drug is taken | 41(4.89) | 488(58.16) | 65(7.75) | 168(20.02) | 53(6.32) | 24(2.86) | 36(3.12) | 713(61.73) | 75(6.49) | 236(20.43) | 66(5.71) | 29(2.51) |
| **Information on adverse reactions** |  |  |  |  |  |  |  |  |  |  |  |  |
| (12) What are the side effects of the drug | 211(25.15) | 400(47.68) | 57(6.79) | 82(9.77) | 26(3.10) | 63(7.51) | 265(22.94) | 583(50.48) | 82(7.10) | 94(8.14) | 32(2.77) | 99(8.57) |
| (13）What will cause the side effects of the drug | 313(37.31) | 329(39.21) | 64(7.63) | 65(7.75) | 22(2.62) | 46(5.48) | 433(37.49) | 484(41.90) | 65(5.63) | 81(7.01) | 32(2.77) | 60(5.19) |
| (14) What to do if you have side effects | 349(41.60) | 333(39.69) | 55(6.56) | 41(4.89) | 27(3.22) | 34(4.05) | 477(41.30) | 499(43.20) | 61(5.28) | 47(4.07) | 30(2.60) | 41(3.55) |
| (15) What to do when you forgot to take the drug or when you took too much | 404(48.15) | 272(32.42) | 62(7.39) | 40(4.77) | 28(3.34) | 33(3.93) | 546(47.27) | 432(37.40) | 70(6.06) | 52(4.50) | 27(2.34) | 28(2.42) |
| (16) What are the effects on your kidney, heart, and life | 336(40.05) | 360(42.91) | 44(5.24) | 53(6.32) | 18(2.15) | 28(3.34) | 501(43.38) | 474(41.04) | 69(5.97) | 55(4.76) | 12(1.04) | 44(3.81) |
| (17) What are the allergies that the drug can cause | 226(26.94) | 419(49.94) | 57(6.79) | 67(7.99) | 35(4.17) | 35(4.17) | 329(28.48) | 592(51.26) | 85(7.36) | 61(5.28) | 27(2.34) | 61(5.28) |
| **Effects on Daily Life** |  |  |  |  |  |  |  |  |  |  |  |  |
| (18) Whether the drug can make you feel drowsy | 304(36.23) | 344(41.00) | 52(6.20) | 58(6.91) | 35(4.17) | 46(5.48) | 424(36.71) | 489(42.34) | 64(5.54) | 60(5.19) | 48(4.16) | 70(6.06) |
| (19) Whether you can drink alcohol whilst taking the drug | 182(21.69) | 474(56.50) | 48(5.72) | 65(7.75) | 33(3.93) | 37(4.41) | 313(27.10) | 594(51.43) | 59(5.11) | 68(5.89) | 48(4.16) | 73(6.32) |
| (20) What are the impacts on driving when taking the  drug | 402(47.91) | 286(34.09) | 42(5.01) | 53(6.32) | 26(3.10) | 30(3.58) | 623(53.94) | 364(31.52) | 50(4.33) | 49(4.24) | 22(1.90) | 47(4.07) |
| (21) What are the impacts on your reaction ability  when taking the drug | 439(52.32) | 253(30.15) | 53(6.32) | 47(5.60) | 23(2.74) | 24(2.86) | 660(57.14) | 330(28.57) | 55(4.76) | 47(4.07) | 26(2.25) | 37(3.20) |
| (22) What are the impacts on your diet when taking  the drug | 241(28.72) | 421(50.18) | 50(5.96) | 63(7.51) | 40(4.77) | 24(2.86) | 322(27.88) | 616(53.33) | 65(5.63) | 63(5.45) | 53(4.59) | 36(3.12) |
| (23) Whether the drug will affect your sex life | 567(67.58) | 166(19.79) | 35(4.17) | 34(4.05) | 11(1.31) | 26(3.10) | 804(69.61) | 232(20.09) | 41(3.55) | 27(2.34) | 16(1.39) | 35(3.03) |
| **Other information** |  |  |  |  |  |  |  |  |  |  |  |  |
| (24) How to get more drugs | 343(40.88) | 266(31.70) | 50(5.96) | 126(15.02) | 23(2.74) | 31(3.69) | 528(45.71) | 386(33.42) | 44(3.81) | 133(11.52) | 19(1.65) | 45(3.90) |
| (25) Whether the drug is reimbursed | 263(31.35) | 316(37.66) | 38(4.53) | 115(13.71) | 22(2.62) | 85(10.13) | 403(34.89) | 380(32.90) | 39(3.38) | 179(15.50) | 36(3.12) | 118(10.22) |
| (26) What are the reasons for different prescriptions when suffering from same illness | 397(47.32) | 346(41.24) | 37(4.41) | 33(3.93) | 10(1.19) | 16(1.91) | 563(48.74) | 487(42.16) | 43(3.72) | 32(2.77) | 9(0.78) | 21(1.82) |
| (27) How to preserve the drug | 302(36.00) | 238(28.37) | 40(4.77) | 153(18.24) | 42(5.01) | 64(7.63) | 391(33.85) | 338(29.26) | 45(3.90) | 211(18.27) | 43(3.72) | 127(11.00) |

| **Table 4 Drug Information Needs of Patients by Sex (n(%))** | | | | | | | | | | |
| --- | --- | --- | --- | --- | --- | --- | --- | --- | --- | --- |
|  | **Male(N=839)** |  |  |  |  | **Female(N=1155)** | |  |  |  |
|  | **Very Unimportant** | **Unimportant** | **Difficult to Judge** | **Important** | **Very Important** | **Very Unimportant** | **Unimportant** | **Difficult to Judge** | **Important** | **Very Important** |
| **General information** |  |  |  |  |  |  |  |  |  |  |
| (1) What is the name of the drug | 6(0.72) | 73(8.70) | 110(13.11) | 435(51.85) | 215(25.63) | 11(0.95) | 106(9.18) | 122(10.56) | 578(50.04) | 338(29.26) |
| (2) What is the expiration date of the drug | 2(0.24) | 48(5.72) | 93(11.08) | 401(47.79) | 295(35.16) | 6(0.52) | 62(5.37) | 85(7.36) | 555(48.05) | 447(38.70) |
| (3) What the drug is for | 2(0.24) | 25(2.98) | 84(10.01) | 438(52.21) | 290(34.56) | 4(0.35) | 25(2.16) | 64(5.54) | 627(54.29) | 435(37.66) |
| (4) How the drug works | 36(4.29) | 219(26.10) | 203(24.20) | 250(29.80) | 131(15.61) | 51(4.42) | 310(26.84) | 269(23.29) | 343(29.70) | 182(15.76) |
| (5) How long does it takes before the drug works | 7(0.83) | 75(8.94) | 109(12.99) | 452(53.87) | 196(23.36) | 8(0.69) | 85(7.36) | 143(12.38) | 620(53.68) | 299(25.89) |
| (6) How to judge if the drug works | 1(0.12) | 68(8.10) | 115(13.71) | 430(51.25) | 225(26.82) | 9(0.78) | 65(5.63) | 164(14.20) | 610(52.81) | 307(26.58) |
| (7) Whether, and if so what the alternatives are for the drug | 17(2.03) | 158(18.83) | 208(24.79) | 336(40.05) | 120(14.30) | 19(1.65) | 200(17.32) | 296(25.63) | 465(40.26) | 175(15.15) |
| (8) Whether there are any interactions with other drugs | 7(0.83) | 33(3.93) | 93(11.08) | 375(44.70) | 331(39.45) | 12(1.04) | 37(3.20) | 90(7.79) | 547(47.36) | 469(40.61) |
| **Usage Information** |  |  |  |  |  |  |  |  |  |  |
| (9) How to use the drug | 3(0.36) | 21(2.50) | 36(4.29) | 453(53.99) | 326(38.86) | 2(0.17) | 18(1.56) | 43(3.72) | 573(49.61) | 519(44.94) |
| (10) How long the drug should be taken | 1(0.12) | 22(2.62) | 56(6.67) | 454(54.11) | 306(36.47) | 3(0.26) | 25(2.16) | 48(4.16) | 625(54.11) | 454(39.31) |
| (11) How much dose the drug is taken | 0(0.00) | 11(1.31) | 46(5.48) | 414(49.34) | 368(43.86) | 3(0.26) | 11(0.95) | 35(3.03) | 580(50.22) | 526(45.54) |
| **Information on adverse reactions** |  |  |  |  |  |  |  |  |  |  |
| (12) What are the side effects of the drug | 3(0.36) | 33(3.93) | 81(9.65) | 434(51.73) | 288(34.33) | 5(0.43) | 34(2.94) | 92(7.97) | 593(51.34) | 431(37.32) |
| (13）What will cause the side effects of the drug | 7(0.83) | 54(6.44) | 128(15.26) | 402(47.91) | 248(29.56) | 4(0.35) | 60(5.19) | 148(12.81) | 549(47.53) | 394(34.11) |
| (14) What to do if you have side effects | 2(0.24) | 49(5.84) | 126(15.02) | 377(44.93) | 285(33.97) | 4(0.35) | 38(3.29) | 133(11.52) | 554(47.97) | 426(36.88) |
| (15) What to do when you forgot to take the drug or when you took too much | 11(1.31) | 64(7.63) | 160(19.07) | 372(44.34) | 232(27.65) | 5(0.43) | 76(6.58) | 192(16.62) | 520(45.02) | 362(31.34) |
| (16) What are the effects on your kidney, heart, and life | 6(0.72) | 36(4.29) | 119(14.18) | 342(40.76) | 336(40.05) | 6(0.52) | 48(4.16) | 142(12.29) | 488(42.25) | 471(40.78) |
| (17) What are the allergies that the drug can cause | 7(0.83) | 36(4.29) | 95(11.32) | 403(48.03) | 298(35.52) | 7(0.61) | 38(3.29) | 111(9.61) | 558(48.31) | 441(38.18) |
| **Effects on Daily Life** |  |  |  |  |  |  |  |  |  |  |
| (18) Whether the drug can make you feel drowsy | 14(1.67) | 146(17.40) | 148(17.64) | 371(44.22) | 160(19.07) | 18(1.56) | 209(18.10) | 162(14.03) | 521(45.11) | 245(21.21) |
| (19) Whether you can drink alcohol whilst taking the drug | 21(2.50) | 85(10.13) | 84(10.01) | 390(46.48) | 259(30.87) | 41(3.55) | 129(11.17) | 106(9.18) | 497(43.03) | 382(33.07) |
| (20) What are the impacts on driving when taking the  drug | 47(5.60) | 114(13.59) | 153(18.24) | 294(35.04) | 231(27.53) | 65(5.63) | 179(15.50) | 195(16.88) | 418(36.19) | 298(25.80) |
| (21) What are the impacts on your reaction ability  when taking the drug | 27(3.22) | 126(15.02) | 196(23.36) | 318(37.90) | 172(20.50) | 23(1.99) | 189(16.36) | 269(23.29) | 425(36.80) | 249(21.56) |
| (22) What are the impacts on your diet when taking  the drug | 16(1.91) | 95(11.32) | 141(16.81) | 400(47.68) | 187(22.29) | 8(0.69) | 126(10.91) | 143(12.38) | 593(51.34) | 285(24.68) |
| (23) Whether the drug will affect your sex life | 83(9.89) | 182(21.69) | 271(32.30) | 195(23.24) | 108(12.87) | 116(10.04) | 301(26.06) | 339(29.35) | 251(21.73) | 148(12.81) |
| **Other information** |  |  |  |  |  |  |  |  |  |  |
| (24) How to get more drugs | 33(3.93) | 178(21.22) | 189(22.53) | 319(38.02) | 120(14.30) | 30(2.60) | 245(21.21) | 251(21.73) | 437(37.84) | 192(16.62) |
| (25) Whether the drug is reimbursed | 13(1.55) | 97(11.56) | 115(13.71) | 406(48.39) | 208(24.79) | 16(1.39) | 132(11.43) | 140(12.12) | 587(50.82) | 280(24.24) |
| (26) What are the reasons for different prescriptions when suffering from same illness | 25(2.98) | 163(19.43) | 209(24.91) | 303(36.11) | 139(16.57) | 25(2.16) | 225(19.48) | 271(23.46) | 438(37.92) | 196(16.97) |
| (27) How to preserve the drug | 11(1.31) | 109(12.99) | 131(15.61) | 427(50.89) | 161(19.19) | 17(1.47) | 138(11.95) | 141(12.21) | 593(51.34) | 266(23.03) |

| **Table 5 Drug Information Obtainment of Patients by Diagnosis and Treatment Status (n(%))** | | | | | | | | | | | | |
| --- | --- | --- | --- | --- | --- | --- | --- | --- | --- | --- | --- | --- |
|  | **Outpatient(N=1274)** | |  |  |  |  | **Inpatient(N=720)** | |  |  |  |  |
|  | **Not Obtained** | **Doctor** | **Clinical Pharmacist** | **Pharmacy- based**  **pharmacist** | **Nurse** | **Others** | **Not Obtained** | **Doctor** | **Clinical Pharmacist** | **Pharmacy- based**  **pharmacist** | **Nurse** | **Others** |
| **General information** |  |  |  |  |  |  |  |  |  |  |  |  |
| (1) What is the name of the drug | 69(5.42) | 862(67.66) | 58(4.55) | 189(14.84) | 31(2.43) | 65(5.10) | 53(7.36) | 454(63.06) | 67(9.31) | 57(7.92) | 59(8.19) | 30(4.17) |
| (2) What is the expiration date of the drug | 360(28.26) | 258(20.25) | 48(3.77) | 229(17.97) | 37(2.90) | 342(26.84) | 231(32.08) | 154(21.39) | 48(6.67) | 93(12.92) | 51(7.08) | 143(19.86) |
| (3) What the drug is for | 127(9.97) | 896(70.33) | 62(4.87) | 110(8.63) | 26(2.04) | 53(4.16) | 77(10.69) | 460(63.89) | 88(12.22) | 38(5.28) | 36(5.00) | 21(2.92) |
| (4) How the drug works | 781(61.30) | 292(22.92) | 51(4.00) | 70(5.49) | 12(0.94) | 68(5.34) | 406(56.39) | 159(22.08) | 76(10.56) | 31(4.31) | 24(3.33) | 24(3.33) |
| (5) How long does it takes before the drug works | 345(27.08) | 704(55.26) | 60(4.71) | 86(6.75) | 32(2.51) | 47(3.69) | 160(22.22) | 355(49.31) | 82(11.39) | 46(6.39) | 54(7.50) | 23(3.19) |
| (6) How to judge if the drug works | 383(30.06) | 606(47.57) | 60(4.71) | 82(6.44) | 22(1.73) | 121(9.50) | 187(25.97) | 333(46.25) | 66(9.17) | 32(4.44) | 51(7.08) | 51(7.08) |
| (7) Whether, and if so what the alternatives are for the drug | 694(54.47) | 372(29.20) | 62(4.87) | 104(8.16) | 13(1.02) | 29(2.28) | 350(48.61) | 217(30.14) | 76(10.56) | 45(6.25) | 13(1.81) | 19(2.64) |
| (8) Whether there are any interactions with other drugs | 316(24.80) | 664(52.12) | 61(4.79) | 166(13.03) | 7(0.55) | 60(4.71) | 182(25.28) | 344(47.78) | 79(10.97) | 64(8.89) | 30(4.17) | 21(2.92) |
| **Usage Information** |  |  |  |  |  |  |  |  |  |  |  |  |
| (9) How to use the drug | 61(4.79) | 732(57.46) | 55(4.32) | 344(27.00) | 41(3.22) | 41(3.22) | 20(2.78) | 377(52.36) | 79(10.97) | 110(15.28) | 106(14.72) | 28(3.89) |
| (10) How long the drug should be taken | 102(8.01) | 902(70.80) | 54(4.24) | 157(12.32) | 26(2.04) | 33(2.59) | 41(5.69) | 476(66.11) | 67(9.31) | 57(7.92) | 62(8.61) | 17(2.36) |
| (11) How much dose the drug is taken | 44(3.45) | 783(61.46) | 63(4.95) | 308(24.18) | 42(3.30) | 34(2.67) | 33(4.58) | 418(58.06) | 77(10.69) | 96(13.33) | 77(10.69) | 19(2.64) |
| **Information on adverse reactions** |  |  |  |  |  |  |  |  |  |  |  |  |
| (12) What are the side effects of the drug | 318(24.96) | 635(49.84) | 62(4.87) | 124(9.73) | 18(1.41) | 117(9.18) | 158(21.94) | 348(48.33) | 77(10.69) | 52(7.22) | 40(5.56) | 45(6.25) |
| (13）What will cause the side effects of the drug | 502(39.40) | 514(40.35) | 56(4.40) | 110(8.63) | 19(1.49) | 73(5.73) | 244(33.89) | 299(41.53) | 73(10.14) | 36(5.00) | 35(4.86) | 33(4.58) |
| (14) What to do if you have side effects | 562(44.11) | 528(41.44) | 50(3.92) | 60(4.71) | 20(1.57) | 54(4.24) | 264(36.67) | 304(42.22) | 66(9.17) | 28(3.89) | 37(5.14) | 21(2.92) |
| (15) What to do when you forgot to take the drug or when you took too much | 661(51.88) | 432(33.91) | 66(5.18) | 66(5.18) | 11(0.86) | 38(2.98) | 289(40.14) | 272(37.78) | 66(9.17) | 26(3.61) | 44(6.11) | 23(3.19) |
| (16) What are the effects on your kidney, heart, and life | 588(46.15) | 499(39.17) | 56(4.40) | 74(5.81) | 7(0.55) | 50(3.92) | 249(34.58) | 335(46.53) | 57(7.92) | 34(4.72) | 23(3.19) | 22(3.06) |
| (17) What are the allergies that the drug can cause | 375(29.43) | 660(51.81) | 60(4.71) | 88(6.91) | 23(1.81) | 68(5.34) | 180(25.00) | 351(48.75) | 82(11.39) | 40(5.56) | 39(5.42) | 28(3.89) |
| **Effects on Daily Life** |  |  |  |  |  |  |  |  |  |  |  |  |
| (18) Whether the drug can make you feel drowsy | 487(38.23) | 541(42.46) | 55(4.32) | 87(6.83) | 29(2.28) | 75(5.89) | 241(33.47) | 292(40.56) | 61(8.47) | 31(4.31) | 54(7.50) | 41(5.69) |
| (19) Whether you can drink alcohol whilst taking the drug | 331(25.98) | 696(54.63) | 49(3.85) | 95(7.46) | 25(1.96) | 78(6.12) | 164(22.78) | 372(51.67) | 58(8.06) | 38(5.28) | 56(7.78) | 32(4.44) |
| (20) What are the impacts on driving when taking the  drug | 677(53.14) | 407(31.95) | 45(3.53) | 72(5.65) | 16(1.26) | 57(4.47) | 348(48.33) | 243(33.75) | 47(6.53) | 30(4.17) | 32(4.44) | 20(2.78) |
| (21) What are the impacts on your reaction ability  when taking the drug | 746(58.56) | 348(27.32) | 52(4.08) | 67(5.26) | 18(1.41) | 43(3.38) | 353(49.03) | 235(32.64) | 56(7.78) | 27(3.75) | 31(4.31) | 18(2.50) |
| (22) What are the impacts on your diet when taking  the drug | 395(31.00) | 667(52.35) | 53(4.16) | 94(7.38) | 25(1.96) | 40(3.14) | 168(23.33) | 370(51.39) | 62(8.61) | 32(4.44) | 68(9.44) | 20(2.78) |
| (23) Whether the drug will affect your sex life | 909(71.35) | 243(19.07) | 35(2.75) | 43(3.38) | 10(0.78) | 34(2.67) | 462(64.17) | 155(21.53) | 41(5.69) | 18(2.50) | 17(2.36) | 27(3.75) |
| **Other information** |  |  |  |  |  |  |  |  |  |  |  |  |
| (24) How to get more drugs | 579(45.45) | 419(32.89) | 40(3.14) | 170(13.34) | 15(1.18) | 51(4.00) | 292(40.56) | 233(32.36) | 54(7.50) | 89(12.36) | 27(3.75) | 25(3.47) |
| (25) Whether the drug is reimbursed | 484(37.99) | 405(31.79) | 35(2.75) | 197(15.46) | 33(2.59) | 120(9.42) | 182(25.28) | 291(40.42) | 42(5.83) | 97(13.47) | 25(3.47) | 83(11.53) |
| (26) What are the reasons for different prescriptions when suffering from same illness | 629(49.37) | 531(41.68) | 40(3.14) | 45(3.53) | 8(0.63) | 21(1.65) | 331(45.97) | 302(41.94) | 40(5.56) | 20(2.78) | 11(1.53) | 16(2.22) |
| (27) How to preserve the drug | 460(36.11) | 352(27.63) | 38(2.98) | 264(20.72) | 29(2.28) | 131(10.28) | 233(32.36) | 224(31.11) | 47(6.53) | 100(13.89) | 56(7.78) | 60(8.33) |

| **Table 6 Drug Information Needs of Patients by Diagnosis and Treatment Status (n(%))** | | | | | | | | | | |
| --- | --- | --- | --- | --- | --- | --- | --- | --- | --- | --- |
|  | **Outpatient(N=1274)** | |  |  |  | **Inpatient(N=720)** | |  |  |  |
|  | **Very Unimportant** | **Unimportant** | **Difficult to Judge** | **Important** | **Very Important** | **Very**  **Unimportant** | **Unimportant** | **Difficult to Judge** | **Important** | **Very Important** |
| **General information** |  |  |  |  |  |  |  |  |  |  |
| (1) What is the name of the drug | 9(0.71) | 115(9.03) | 165(12.95) | 649(50.94) | 336(26.37) | 8(1.11) | 64(8.89) | 67(9.31) | 364(50.56) | 217(30.14) |
| (2) What is the expiration date of the drug | 7(0.55) | 71(5.57) | 116(9.11) | 606(47.57) | 474(37.21) | 1(0.14) | 39(5.42) | 62(8.61) | 350(48.61) | 268(37.22) |
| (3) What the drug is for | 5(0.39) | 33(2.59) | 96(7.54) | 692(54.32) | 448(35.16) | 1(0.14) | 17(2.36) | 52(7.22) | 373(51.81) | 277(38.47) |
| (4) How the drug works | 55(4.32) | 355(27.86) | 308(24.18) | 368(28.89) | 188(14.76) | 32(4.44) | 174(24.17) | 164(22.78) | 225(31.25) | 125(17.36) |
| (5) How long does it takes before the drug works | 12(0.94) | 108(8.48) | 159(12.48) | 694(54.47) | 301(23.63) | 3(0.42) | 52(7.22) | 93(12.92) | 378(52.50) | 194(26.94) |
| (6) How to judge if the drug works | 9(0.71) | 91(7.14) | 176(13.81) | 673(52.83) | 325(25.51) | 1(0.14) | 42(5.83) | 103(14.31) | 367(50.97) | 207(28.75) |
| (7) Whether, and if so what the alternatives are for the drug | 22(1.73) | 235(18.45) | 330(25.90) | 514(40.35) | 173(13.58) | 14(1.94) | 123(17.08) | 174(24.17) | 287(39.86) | 122(16.94) |
| (8) Whether there are any interactions with other drugs | 14(1.10) | 38(2.98) | 118(9.26) | 589(46.23) | 515(40.42) | 5(0.69) | 32(4.44) | 65(9.03) | 333(46.25) | 285(39.58) |
| **Usage Information** |  |  |  |  |  |  |  |  |  |  |
| (9) How to use the drug | 3(0.24) | 21(1.65) | 49(3.85) | 654(51.33) | 547(42.94) | 2(0.28) | 18(2.50) | 30(4.17) | 372(51.67) | 298(41.39) |
| (10) How long the drug should be taken | 3(0.24) | 30(2.35) | 64(5.02) | 696(54.63) | 481(37.76) | 1(0.14) | 17(2.36) | 40(5.56) | 383(53.19) | 279(38.75) |
| (11) How much dose the drug is taken | 3(0.24) | 14(1.10) | 49(3.85) | 621(48.74) | 587(46.08) | 0(0.00) | 8(1.11) | 32(4.44) | 373(51.81) | 307(42.64) |
| **Information on adverse reactions** |  |  |  |  |  |  |  |  |  |  |
| (12) What are the side effects of the drug | 6(0.47) | 50(3.92) | 115(9.03) | 643(50.47) | 460(36.11) | 2(0.28) | 17(2.36) | 58(8.06) | 384(53.33) | 259(35.97) |
| (13）What will cause the side effects of the drug | 10(0.78) | 72(5.65) | 187(14.68) | 598(46.94) | 407(31.95) | 1(0.14) | 42(5.83) | 89(12.36) | 353(49.03) | 235(32.64) |
| (14) What to do if you have side effects | 5(0.39) | 58(4.55) | 173(13.58) | 588(46.15) | 450(35.32) | 1(0.14) | 29(4.03) | 86(11.94) | 343(47.64) | 261(36.25) |
| (15) What to do when you forgot to take the drug or when you took too much | 9(0.71) | 85(6.67) | 236(18.52) | 576(45.21) | 368(28.89) | 7(0.97) | 55(7.64) | 116(16.11) | 316(43.89) | 226(31.39) |
| (16) What are the effects on your kidney, heart, and life | 9(0.71) | 61(4.79) | 175(13.74) | 532(41.76) | 497(39.01) | 3(0.42) | 23(3.19) | 86(11.94) | 298(41.39) | 310(43.06) |
| (17) What are the allergies that the drug can cause | 11(0.86) | 48(3.77) | 132(10.36) | 616(48.35) | 467(36.66) | 3(0.42) | 26(3.61) | 74(10.28) | 345(47.92) | 272(37.78) |
| **Effects on Daily Life** |  |  |  |  |  |  |  |  |  |  |
| (18) Whether the drug can make you feel drowsy | 21(1.65) | 228(17.90) | 194(15.23) | 574(45.05) | 257(20.17) | 11(1.53) | 127(17.64) | 116(16.11) | 318(44.17) | 148(20.56) |
| (19) Whether you can drink alcohol whilst taking the drug | 36(2.83) | 132(10.36) | 121(9.50) | 578(45.37) | 407(31.95) | 26(3.61) | 82(11.39) | 69(9.58) | 309(42.92) | 234(32.50) |
| (20) What are the impacts on driving when taking the  drug | 66(5.18) | 177(13.89) | 232(18.21) | 457(35.87) | 342(26.84) | 46(6.39) | 116(16.11) | 116(16.11) | 255(35.42) | 187(25.97) |
| (21) What are the impacts on your reaction ability  when taking the drug | 32(2.51) | 193(15.15) | 301(23.63) | 475(37.28) | 273(21.43) | 18(2.50) | 122(16.94) | 164(22.78) | 268(37.22) | 148(20.56) |
| (22) What are the impacts on your diet when taking  the drug | 16(1.26) | 158(12.40) | 183(14.36) | 628(49.29) | 289(22.68) | 8(1.11) | 63(8.75) | 101(14.03) | 365(50.69) | 183(25.42) |
| (23) Whether the drug will affect your sex life | 125(9.81) | 299(23.47) | 412(32.34) | 285(22.37) | 153(12.01) | 74(10.28) | 184(25.56) | 198(27.50) | 161(22.36) | 103(14.31) |
| **Other information** |  |  |  |  |  |  |  |  |  |  |
| (24) How to get more drugs | 39(3.06) | 277(21.74) | 301(23.63) | 477(37.44) | 180(14.13) | 24(3.33) | 146(20.28) | 139(19.31) | 279(38.75) | 132(18.33) |
| (25) Whether the drug is reimbursed | 24(1.88) | 163(12.79) | 178(13.97) | 641(50.31) | 268(21.04) | 5(0.69) | 66(9.17) | 77(10.69) | 352(48.89) | 220(30.56) |
| (26) What are the reasons for different prescriptions when suffering from same illness | 34(2.67) | 246(19.31) | 335(26.30) | 460(36.11) | 199(15.62) | 16(2.22) | 142(19.72) | 145(20.14) | 281(39.03) | 136(18.89) |
| (27) How to preserve the drug | 16(1.26) | 153(12.01) | 176(13.81) | 677(53.14) | 252(19.78) | 12(1.67) | 94(13.06) | 96(13.33) | 343(47.64) | 175(24.31) |

| **Table 7 Drug Information Obtainment of Patients by Level of Education (n(%))** | | | | | | | | | | | | | | | | | | | | | | | | | | | | | | | | | | | | |
| --- | --- | --- | --- | --- | --- | --- | --- | --- | --- | --- | --- | --- | --- | --- | --- | --- | --- | --- | --- | --- | --- | --- | --- | --- | --- | --- | --- | --- | --- | --- | --- | --- | --- | --- | --- | --- |
|  | **Primary School Education or Below (N=205)** | | | |  |  | **Junior High School Education(N=314)** | | | |  |  | **High School Education/Secondary School Education(N=411)** | | | | | | **College Degree(N=321)** | |  |  |  |  | **Bachelor Degree(N=701)** | | |  |  |  | **Master Degree or Above(N=42)** | | | |  |  |
|  | **Not Obtained** | **Doctor** | **Clinical Pharma cist** | **Pharmacy- based pharmacist** | **Nurse** | **Others** | **Not Obtain** | **Doctor** | **Clinical P** | **Pharma** | **Nurse** | **Others** | **Not Obtain** | **Doctor** | **Clinical** | **Pharmacy** | **Nurse** | **Others** | **Not Obtained** | **Doctor** | **Clinical Pharmacist** | **Pharma cy-based pharmacist** | **Nurse** | **Others** | **Not Obtained** | **Doctor** | **Clinical Pharma cist** | **Pharma cy-based pharmacist** | **Nurse** | **Others** | **Not Obtained** | **Doctor** | **Clinical Pharma cist** | **Pharmacy-based pharmacist** | **Nurse** | **Others** |
| **General information** |  |  |  |  |  |  |  |  |  |  |  |  |  |  |  |  |  |  |  |  |  |  |  |  |  |  |  |  |  |  |  |  |  |  |  |  |
| (1) What is the name of the drug | 26(12.68) | 126(61.46) | 8(3.90) | 19(9.27) | 13(6.34) | 13(6.34) | 27(8.60) | 206(65.61) | 28(8.92) | 26(8.28) | 17(5.41) | 10(3.18) | 20(4.87) | 278(67.64) | 23(5.60) | 49(11.92) | 22(5.35) | 19(4.62) | 20(6.23) | 214(66.67) | 21(6.54) | 40(12.46) | 14(4.36) | 12(3.74) | 27(3.85) | 471(67.19) | 41(5.85) | 107(15.26 | 22(3.14) | 33(4.71) | 2(4.76) | 21(50.00) | 4(9.52) | 5(11.90) | 2(4.76) | 8(19.05) |
| (2) What is the expiration date of the  drug | 82(40.00) | 51(24.88) | 9(4.39) | 21(10.24) | 10(4.88) | 32(15.61) | 104(33.12) | 77(24.52) | 16(5.10) | 36(11.46 | 15(4.78) | 66(21.02 | 128(31.14) | 71(17.27) | 20(4.87) | 60(14.60) | 25(6.08) | 107(26.03) | 95(29.60) | 59(18.38) | 16(4.98) | 57(17.76) | 13(4.05) | 81(25.23 | 173(24.68) | 148(21.11) | 33(4.71) | 142(20.26 | 24(3.42) | 181(25.82 | 9(21.43) | 6(14.29) | 2(4.76) | 6(14.29) | 1(2.38) | 18(42.86) |
| (3) What the drug is for | 27(13.17) | 135(65.85) | 12(5.85) | 15(7.32) | 9(4.39) | 7(3.41) | 41(13.06) | 208(66.24) | 28(8.92) | 19(6.05) | 7(2.23) | 11(3.50) | 49(11.92) | 272(66.18) | 30(7.30) | 22(5.35) | 17(4.14) | 21(5.11) | 27(8.41) | 224(69.78) | 27(8.41) | 23(7.17) | 9(2.80) | 11(3.43) | 58(8.27) | 493(70.33) | 47(6.70) | 66(9.42) | 19(2.71) | 18(2.57) | 2(4.76) | 24(57.14) | 6(14.29) | 3(7.14) | 1(2.38) | 6(14.29) |
| (4) How the drug works | 132(64.39) | 41(20.00) | 15(7.32) | 7(3.41) | 4(1.95) | 6(2.93) | 190(60.51) | 75(23.89) | 26(8.28) | 7(2.23) | 8(2.55) | 8(2.55) | 246(59.85) | 83(20.19) | 28(6.81) | 22(5.35) | 9(2.19) | 23(5.60) | 188(58.57) | 78(24.30) | 20(6.23) | 23(7.17) | 3(0.93) | 9(2.80) | 409(58.35) | 168(23.97) | 34(4.85) | 40(5.71) | 11(1.57) | 39(5.56) | 22(52.38) | 6(14.29) | 4(9.52) | 2(4.76) | 1(2.38) | 7(16.67) |
| (5) How long does it takes before the  drug works | 57(27.80) | 112(54.63) | 14(6.83) | 9(4.39) | 6(2.93) | 7(3.41) | 81(25.80) | 169(53.82) | 24(7.64) | 15(4.78) | 16(5.10) | 9(2.87) | 113(27.49) | 198(48.18) | 31(7.54) | 29(7.06) | 28(6.81) | 12(2.92) | 73(22.74) | 188(58.57) | 20(6.23) | 22(6.85) | 7(2.18) | 11(3.43) | 167(23.82) | 378(53.92) | 49(6.99) | 53(7.56) | 27(3.85) | 27(3.85) | 14(33.33) | 14(33.33) | 4(9.52) | 4(9.52) | 2(4.76) | 4(9.52) |
| (6) How to judge if the drug works | 59(28.78) | 102(49.76) | 13(6.34) | 14(6.83) | 6(2.93) | 11(5.37) | 100(31.85) | 144(45.86) | 23(7.32) | 9(2.87) | 13(4.14) | 25(7.96) | 114(27.74) | 183(44.53) | 31(7.54) | 25(6.08) | 28(6.81) | 30(7.30) | 92(28.66) | 159(49.53) | 18(5.61) | 23(7.17) | 6(1.87) | 23(7.17) | 189(26.96) | 339(48.36) | 37(5.28) | 41(5.85) | 19(2.71) | 76(10.84) | 16(38.10) | 12(28.57) | 4(9.52) | 2(4.76) | 1(2.38) | 7(16.67) |
| (7) Whether, and if so what the  alternatives are for the drug | 118(57.56) | 54(26.34) | 14(6.83) | 10(4.88) | 3(1.46) | 6(2.93) | 161(51.27) | 99(31.53) | 25(7.96) | 18(5.73) | 6(1.91) | 5(1.59) | 211(51.34) | 114(27.74) | 33(8.03) | 32(7.79) | 12(2.92) | 9(2.19) | 167(52.02) | 99(30.84) | 21(6.54) | 27(8.41) | 0(0.00) | 7(2.18) | 364(51.93) | 218(31.10) | 43(6.13) | 56(7.99) | 4(0.57) | 16(2.28) | 23(54.76) | 5(11.90) | 2(4.76) | 6(14.29) | 1(2.38) | 5(11.90) |
| (8) Whether there are any  interactions with other drugs | 70(34.15) | 95(46.34) | 12(5.85) | 18(8.78) | 6(2.93) | 4(1.95) | 79(25.16) | 152(48.41) | 32(10.19) | 30(9.55) | 8(2.55) | 13(4.14) | 104(25.30) | 202(49.15) | 25(6.08) | 45(10.95) | 14(3.41) | 21(5.11) | 75(23.36) | 167(52.02) | 23(7.17) | 43(13.40) | 1(0.31) | 12(3.74) | 157(22.40) | 375(53.50) | 43(6.13) | 91(12.98) | 8(1.14) | 27(3.85) | 13(30.95) | 17(40.48) | 5(11.90) | 3(7.14) | 0(0.00) | 4(9.52) |
| **Usage Information** |  |  |  |  |  |  |  |  |  |  |  |  |  |  |  |  |  |  |  |  |  |  |  |  |  |  |  |  |  |  |  |  |  |  |  |  |
| (9) How to use the drug | 15(7.32) | 119(58.05) | 12(5.85) | 34(16.59) | 19(9.27) | 6(2.93) | 14(4.46) | 180(57.32) | 26(8.28) | 55(17.52 | 27(8.60) | 12(3.82) | 15(3.65) | 231(56.20) | 29(7.06) | 85(20.68) | 36(8.76) | 15(3.65) | 11(3.43) | 186(57.94) | 23(7.17) | 80(24.92) | 12(3.74) | 9(2.80) | 23(3.28) | 371(52.92) | 39(5.56) | 196(27.96 | 51(7.28) | 21(3.00) | 3(7.14) | 22(52.38) | 5(11.90) | 4(9.52) | 2(4.76) | 6(14.29) |
| (10) How long the drug should be  taken | 21(10.24) | 127(61.95) | 13(6.34) | 21(10.24) | 14(6.83) | 9(4.39) | 30(9.55) | 212(67.52) | 25(7.96) | 24(7.64) | 16(5.10) | 7(2.23) | 27(6.57) | 285(69.34) | 22(5.35) | 39(9.49) | 25(6.08) | 13(3.16) | 17(5.30) | 240(74.77) | 17(5.30) | 34(10.59) | 5(1.56) | 8(2.49) | 43(6.13) | 488(69.61) | 40(5.71) | 94(13.41) | 26(3.71) | 10(1.43) | 5(11.90) | 26(61.90) | 4(9.52) | 2(4.76) | 2(4.76) | 3(7.14) |
| (11) How much dose the drug is  taken | 13(6.34) | 126(61.46) | 11(5.37) | 29(14.15) | 16(7.80) | 10(4.88) | 14(4.46) | 201(64.01) | 21(6.69) | 45(14.33 | 26(8.28) | 7(2.23) | 17(4.14) | 242(58.88) | 30(7.30) | 78(18.98) | 32(7.79) | 12(2.92) | 9(2.80) | 199(61.99) | 25(7.79) | 72(22.43) | 9(2.80) | 7(2.18) | 20(2.85) | 410(58.49) | 47(6.70) | 177(25.25 | 34(4.85) | 13(1.85) | 4(9.52) | 23(54.76) | 6(14.29) | 3(7.14) | 2(4.76) | 4(9.52) |
| **Information on adverse reactions** |  |  |  |  |  |  |  |  |  |  |  |  |  |  |  |  |  |  |  |  |  |  |  |  |  |  |  |  |  |  |  |  |  |  |  |  |
| (12) What are the side effects of the  drug | 65(31.71) | 98(47.80) | 17(8.29) | 8(3.90) | 6(2.93) | 11(5.37) | 75(23.89) | 159(50.64) | 23(7.32) | 20(6.37) | 15(4.78) | 22(7.01) | 109(26.52) | 187(45.50) | 31(7.54) | 33(8.03) | 17(4.14) | 34(8.27) | 67(20.87) | 173(53.89) | 18(5.61) | 37(11.53) | 2(0.62) | 24(7.48) | 149(21.26) | 350(49.93) | 46(6.56) | 74(10.56) | 17(2.43) | 65(9.27) | 11(26.19) | 16(38.10) | 4(9.52) | 4(9.52) | 1(2.38) | 6(14.29) |
| (13）What will cause the side effects of the drug | 90(43.90) | 78(38.05) | 14(6.83) | 8(3.90) | 4(1.95) | 11(5.37) | 118(37.58) | 125(39.81) | 28(8.92) | 18(5.73) | 11(3.50) | 14(4.46) | 167(40.63) | 152(36.98) | 24(5.84) | 30(7.30) | 18(4.38) | 20(4.87) | 114(35.51) | 140(43.61) | 16(4.98) | 32(9.97) | 2(0.62) | 17(5.30) | 241(34.38) | 306(43.65) | 44(6.28) | 55(7.85) | 18(2.57) | 37(5.28) | 16(38.10) | 12(28.57) | 3(7.14) | 3(7.14) | 1(2.38) | 7(16.67) |
| (14) What to do if you have side  effects | 81(39.51) | 88(42.93) | 14(6.83) | 7(3.41) | 4(1.95) | 11(5.37) | 129(41.08) | 128(40.76) | 22(7.01) | 15(4.78) | 13(4.14) | 7(2.23) | 174(42.34) | 163(39.66) | 23(5.60) | 17(4.14) | 18(4.38) | 16(3.89) | 137(42.68) | 142(44.24) | 12(3.74) | 15(4.67) | 4(1.25) | 11(3.43) | 286(40.80) | 297(42.37) | 43(6.13) | 34(4.85) | 15(2.14) | 26(3.71) | 19(45.24) | 14(33.33) | 2(4.76) | 0(0.00) | 3(7.14) | 4(9.52) |
| (15) What to do when you forgot to  take the drug or when you took too much | 90(43.90) | 73(35.61) | 19(9.27) | 7(3.41) | 6(2.93) | 10(4.88) | 148(47.13) | 109(34.71) | 23(7.32) | 13(4.14) | 15(4.78) | 6(1.91) | 195(47.45) | 147(35.77) | 26(6.33) | 20(4.87) | 12(2.92) | 11(2.68) | 147(45.79) | 125(38.94) | 16(4.98) | 16(4.98) | 4(1.25) | 13(4.05) | 348(49.64) | 238(33.95) | 46(6.56) | 33(4.71) | 18(2.57) | 18(2.57) | 22(52.38) | 12(28.57) | 2(4.76) | 3(7.14) | 0(0.00) | 3(7.14) |
| (16) What are the effects on your  kidney, heart, and life | 77(37.56) | 93(45.37) | 16(7.80) | 7(3.41) | 4(1.95) | 8(3.90) | 118(37.58) | 143(45.54) | 22(7.01) | 14(4.46) | 4(1.27) | 13(4.14) | 187(45.50) | 154(37.47) | 21(5.11) | 26(6.33) | 9(2.19) | 14(3.41) | 129(40.19) | 144(44.86) | 17(5.30) | 20(6.23) | 2(0.62) | 9(2.80) | 308(43.94) | 290(41.37) | 33(4.71) | 39(5.56) | 9(1.28) | 22(3.14) | 18(42.86) | 10(23.81) | 4(9.52) | 2(4.76) | 2(4.76) | 6(14.29) |
| (17) What are the allergies that the  drug can cause | 58(28.29) | 105(51.22) | 18(8.78) | 12(5.85) | 3(1.46) | 9(4.39) | 81(25.80) | 164(52.23) | 24(7.64) | 19(6.05) | 14(4.46) | 12(3.82) | 120(29.20) | 201(48.91) | 24(5.84) | 30(7.30) | 18(4.38) | 18(4.38) | 83(25.86) | 168(52.34) | 23(7.17) | 24(7.48) | 6(1.87) | 17(5.30) | 203(28.96) | 354(50.50) | 49(6.99) | 40(5.71) | 20(2.85) | 35(4.99) | 10(23.81) | 19(45.24) | 4(9.52) | 3(7.14) | 1(2.38) | 5(11.90) |
| **Effects on Daily Life** |  |  |  |  |  |  |  |  |  |  |  |  |  |  |  |  |  |  |  |  |  |  |  |  |  |  |  |  |  |  |  |  |  |  |  |  |
| (18) Whether the drug can make you  feel drowsy | 83(40.49) | 81(39.51) | 14(6.83) | 7(3.41) | 9(4.39) | 11(5.37) | 115(36.62) | 129(41.08) | 24(7.64) | 17(5.41) | 15(4.78) | 14(4.46) | 151(36.74) | 164(39.90) | 17(4.14) | 25(6.08) | 23(5.60) | 31(7.54) | 106(33.02) | 155(48.29) | 19(5.92) | 17(5.30) | 6(1.87) | 18(5.61) | 251(35.81) | 295(42.08) | 39(5.56) | 51(7.28) | 27(3.85) | 38(5.42) | 22(52.38) | 9(21.43) | 3(7.14) | 1(2.38) | 3(7.14) | 4(9.52) |
| (19) Whether you can drink alcohol  whilst taking the drug | 75(36.59) | 93(45.37) | 13(6.34) | 7(3.41) | 12(5.85) | 5(2.44) | 73(23.25) | 169(53.82) | 21(6.69) | 22(7.01) | 8(2.55) | 21(6.69) | 102(24.82) | 217(52.80) | 21(5.11) | 24(5.84) | 23(5.60) | 24(5.84) | 58(18.07) | 200(62.31) | 16(4.98) | 20(6.23) | 10(3.12) | 17(5.30) | 172(24.54) | 372(53.07) | 34(4.85) | 58(8.27) | 26(3.71) | 39(5.56) | 15(35.71) | 17(40.48) | 2(4.76) | 2(4.76) | 2(4.76) | 4(9.52) |
| (20) What are the impacts on driving  when taking the drug | 123(60.00) | 54(26.34) | 9(4.39) | 7(3.41) | 5(2.44) | 7(3.41) | 168(53.50) | 95(30.25) | 17(5.41) | 18(5.73) | 4(1.27) | 12(3.82) | 211(51.34) | 135(32.85) | 13(3.16) | 19(4.62) | 18(4.38) | 15(3.65) | 136(42.37) | 135(42.06) | 15(4.67) | 17(5.30) | 4(1.25) | 14(4.36) | 362(51.64) | 222(31.67) | 37(5.28) | 39(5.56) | 16(2.28) | 25(3.57) | 25(59.52) | 9(21.43) | 1(2.38) | 2(4.76) | 1(2.38) | 4(9.52) |
| (21) What are the impacts on your reaction ability when taking the  drug | 115(56.10) | 61(29.76) | 14(6.83) | 8(3.90) | 5(2.44) | 2(0.98) | 178(56.69) | 89(28.34) | 20(6.37) | 15(4.78) | 4(1.27) | 8(2.55) | 227(55.23) | 110(26.76) | 20(4.87) | 23(5.60) | 17(4.14) | 14(3.41) | 166(51.71) | 105(32.71) | 20(6.23) | 11(3.43) | 4(1.25) | 15(4.67) | 388(55.35) | 211(30.10) | 33(4.71) | 36(5.14) | 16(2.28) | 17(2.43) | 25(59.52) | 7(16.67) | 1(2.38) | 1(2.38) | 3(7.14) | 5(11.90) |
| (22) What are the impacts on your  diet when taking the drug | 54(26.34) | 106(51.71) | 18(8.78) | 5(2.44) | 16(7.80) | 6(2.93) | 100(31.85) | 154(49.04) | 19(6.05) | 19(6.05) | 11(3.50) | 11(3.50) | 106(25.79) | 217(52.80) | 19(4.62) | 25(6.08) | 27(6.57) | 17(4.14) | 80(24.92) | 185(57.63) | 18(5.61) | 20(6.23) | 7(2.18) | 11(3.43) | 205(29.24) | 361(51.50) | 39(5.56) | 55(7.85) | 29(4.14) | 12(1.71) | 18(42.86) | 14(33.33) | 2(4.76) | 2(4.76) | 3(7.14) | 3(7.14) |
| (23) Whether the drug will affect  your sex life | 150(73.17) | 30(14.63) | 14(6.83) | 2(0.98) | 2(0.98) | 7(3.41) | 213(67.83) | 67(21.34) | 12(3.82) | 7(2.23) | 4(1.27) | 11(3.50) | 286(69.59) | 75(18.25) | 13(3.16) | 14(3.41) | 10(2.43) | 13(3.16) | 199(61.99) | 85(26.48) | 12(3.74) | 11(3.43) | 1(0.31) | 13(4.05) | 496(70.76) | 134(19.12) | 25(3.57) | 25(3.57) | 8(1.14) | 13(1.85) | 27(64.29) | 7(16.67) | 0(0.00) | 2(4.76) | 2(4.76) | 4(9.52) |
| **Other information** |  |  |  |  |  |  |  |  |  |  |  |  |  |  |  |  |  |  |  |  |  |  |  |  |  |  |  |  |  |  |  |  |  |  |  |  |
| (24) How to get more drugs | 92(44.88) | 71(34.63) | 11(5.37) | 21(10.24) | 3(1.46) | 7(3.41) | 130(41.40) | 107(34.08) | 19(6.05) | 34(10.83 | 12(3.82) | 12(3.82) | 183(44.53) | 132(32.12) | 13(3.16) | 56(13.63) | 11(2.68) | 16(3.89) | 132(41.12) | 110(34.27) | 20(6.23) | 42(13.08) | 3(0.93) | 14(4.36) | 317(45.22) | 223(31.81) | 28(3.99) | 99(14.12) | 10(1.43) | 24(3.42) | 17(40.48) | 9(21.43) | 3(7.14) | 7(16.67) | 3(7.14) | 3(7.14) |
| (25) Whether the drug is reimbursed | 61(29.76) | 82(40.00) | 12(5.85) | 18(8.78) | 3(1.46) | 29(14.15) | 85(27.07) | 122(38.85) | 13(4.14) | 51(16.24 | 7(2.23) | 36(11.46 | 140(34.06) | 142(34.55) | 12(2.92) | 63(15.33) | 18(4.38) | 36(8.76) | 101(31.46) | 110(34.27) | 15(4.67) | 51(15.89) | 5(1.56) | 39(12.15 | 267(38.09) | 228(32.52) | 24(3.42) | 103(14.69 | 22(3.14) | 57(8.13) | 12(28.57) | 12(28.57) | 1(2.38) | 8(19.05) | 3(7.14) | 6(14.29) |
| (26) What are the reasons for different prescriptions when  suffering from same illness | 102(49.76) | 76(37.07) | 10(4.88) | 9(4.39) | 3(1.46) | 5(2.44) | 150(47.77) | 137(43.63) | 12(3.82) | 8(2.55) | 4(1.27) | 3(0.96) | 213(51.82) | 163(39.66) | 14(3.41) | 7(1.70) | 5(1.22) | 9(2.19) | 144(44.86) | 138(42.99) | 17(5.30) | 12(3.74) | 2(0.62) | 8(2.49) | 329(46.93) | 305(43.51) | 26(3.71) | 27(3.85) | 4(0.57) | 10(1.43) | 22(52.38) | 14(33.33) | 1(2.38) | 2(4.76) | 1(2.38) | 2(4.76) |
| (27) How to preserve the drug | 81(39.51) | 69(33.66) | 8(3.90) | 20(9.76) | 10(4.88) | 17(8.29) | 105(33.44) | 104(33.12) | 15(4.78) | 52(16.56 | 13(4.14) | 25(7.96) | 145(35.28) | 111(27.01) | 14(3.41) | 71(17.27) | 27(6.57) | 43(10.46) | 102(31.78) | 95(29.60) | 13(4.05) | 62(19.31) | 8(2.49) | 41(12.77 | 241(34.38) | 191(27.25) | 35(4.99) | 151(21.54 | 25(3.57) | 58(8.27) | 19(45.24) | 6(14.29) | 0(0.00) | 8(19.05) | 2(4.76) | 7(16.67) |

| **Table 8 Drug Information Needs of Patients by Level of Education (n(%))** | | | | | | | | | | | | | | | | | | | | | | | | | | | | | | |
| --- | --- | --- | --- | --- | --- | --- | --- | --- | --- | --- | --- | --- | --- | --- | --- | --- | --- | --- | --- | --- | --- | --- | --- | --- | --- | --- | --- | --- | --- | --- |
|  | **Primary School Education or Below (N=205)** | | | |  | **Junior High School Education(N=314)** | | | |  | **High School Education/Secondary School Education(N=411)** | | | |  | **College Degree(N=321)** | | |  |  | **Bachelor Degree(N=701)** | | |  |  | **Master Degree or Above(N=42)** | | | |  |
|  | **Very Unimportant** | **Unimportant** | **Difficult to Judge** | **Important** | **Very Important** | **Very Unimportant** | **Unimportant** | **Difficult to Judge** | **Important** | **Very Important** | **Very Unimportant** | **Unimportant** | **Difficult to Judge** | **Important** | **Very Important** | **Very Unimportant** | **Unimportant** | **Difficult to Judge** | **Important** | **Very Important** | **Very Unimportant** | **Unimportant** | **Difficult to**  **Judge** | **Important** | **Very Important** | **Very Unimportant** | **Unimportant** | **Difficult to Judge** | **Important** | **Very Important** |
| **General information** |  |  |  |  |  |  |  |  |  |  |  |  |  |  |  |  |  |  |  |  |  |  |  |  |  |  |  |  |  |  |
| (1) What is the name of the drug | 6(2.93) | 22(10.73) | 33(16.10 | 97(47.32) | 47(22.93) | 6(1.91) | 36(11.46) | 32(10.19) | 148(47.13) | 92(29.30 | 0(0.00) | 35(8.52) | 44(10.71) | 209(50.85) | 123(29.9 | 1(0.31) | 18(5.61) | 33(10.28) | 181(56.39) | 88(27.41) | 4(0.57) | 65(9.27) | 86(12.27 | 357(50.9 | 189(26.96) | 0(0.00) | 3(7.14) | 4(9.52) | 21(50.00 | 14(33.33) |
| (2) What is the expiration date of the  drug | 0(0.00) | 14(6.83) | 35(17.07 | 86(41.95) | 70(34.15) | 2(0.64) | 25(7.96) | 23(7.32) | 142(45.22) | 122(38.8 | 2(0.49) | 27(6.57) | 31(7.54) | 185(45.01) | 166(40.3 | 3(0.93) | 16(4.98) | 27(8.41) | 159(49.53) | 116(36.14) | 1(0.14) | 25(3.57) | 59(8.42) | 364(51.9 | 252(35.95) | 0(0.00) | 3(7.14) | 3(7.14) | 20(47.62 | 16(38.10) |
| (3) What the drug is for | 0(0.00) | 10(4.88) | 25(12.20 | 92(44.88) | 78(38.05) | 2(0.64) | 9(2.87) | 22(7.01) | 164(52.23) | 117(37.2 | 0(0.00) | 10(2.43) | 36(8.76) | 218(53.04) | 147(35.7 | 2(0.62) | 7(2.18) | 19(5.92) | 173(53.89) | 120(37.38) | 2(0.29) | 14(2.00) | 45(6.42) | 395(56.3 | 245(34.95) | 0(0.00) | 0(0.00) | 1(2.38) | 23(54.76 | 18(42.86) |
| (4) How the drug works | 13(6.34) | 43(20.98) | 58(28.29 | 57(27.80) | 34(16.59) | 20(6.37) | 89(28.34) | 63(20.06) | 88(28.03) | 54(17.20 | 11(2.68) | 118(28.7 | 107(26.03) | 113(27.49) | 62(15.09 | 10(3.12) | 79(24.61) | 75(23.36) | 97(30.22) | 60(18.69) | 33(4.71) | 189(26.96 | 162(23.1 | 220(31.3 | 97(13.84) | 0(0.00) | 11(26.19 | 7(16.67) | 18(42.86 | 6(14.29) |
| (5) How long does it takes before the  drug works | 1(0.49) | 14(6.83) | 35(17.07 | 99(48.29) | 56(27.32) | 3(0.96) | 31(9.87) | 41(13.06) | 152(48.41) | 87(27.71 | 3(0.73) | 42(10.22 | 56(13.63) | 216(52.55) | 94(22.87 | 2(0.62) | 21(6.54) | 38(11.84) | 171(53.27) | 89(27.73) | 5(0.71) | 45(6.42) | 77(10.98 | 415(59.2 | 159(22.68) | 1(2.38) | 7(16.67) | 5(11.90) | 19(45.24 | 10(23.81) |
| (6) How to judge if the drug works | 1(0.49) | 15(7.32) | 31(15.12 | 107(52.20) | 51(24.88) | 5(1.59) | 22(7.01) | 44(14.01) | 161(51.27) | 82(26.11 | 0(0.00) | 36(8.76) | 55(13.38) | 208(50.61) | 112(27.2 | 1(0.31) | 19(5.92) | 43(13.40) | 168(52.34) | 90(28.04) | 3(0.43) | 38(5.42) | 98(13.98 | 378(53.9 | 184(26.25) | 0(0.00) | 3(7.14) | 8(19.05) | 18(42.86 | 13(30.95) |
| (7) Whether, and if so what the  alternatives are for the drug | 5(2.44) | 33(16.10) | 68(33.17 | 69(33.66) | 30(14.63) | 5(1.59) | 64(20.38) | 70(22.29) | 120(38.22) | 55(17.52 | 8(1.95) | 87(21.17 | 92(22.38) | 158(38.44) | 66(16.06 | 6(1.87) | 46(14.33) | 82(25.55) | 130(40.50) | 57(17.76) | 11(1.57) | 119(16.98 | 181(25.8 | 309(44.0 | 81(11.55) | 1(2.38) | 9(21.43) | 11(26.19) | 15(35.71 | 6(14.29) |
| (8) Whether there are any  interactions with other drugs | 2(0.98) | 11(5.37) | 36(17.56 | 87(42.44) | 69(33.66) | 6(1.91) | 12(3.82) | 31(9.87) | 136(43.31) | 129(41.0 | 3(0.73) | 19(4.62) | 39(9.49) | 183(44.53) | 167(40.6 | 3(0.93) | 8(2.49) | 26(8.10) | 149(46.42) | 135(42.06) | 4(0.57) | 19(2.71) | 47(6.70) | 346(49.3 | 285(40.66) | 1(2.38) | 1(2.38) | 4(9.52) | 21(50.00 | 15(35.71) |
| **Usage Information** |  |  |  |  |  |  |  |  |  |  |  |  |  |  |  |  |  |  |  |  |  |  |  |  |  |  |  |  |  |  |
| (9) How to use the drug | 1(0.49) | 5(2.44) | 15(7.32) | 97(47.32) | 87(42.44) | 2(0.64) | 6(1.91) | 10(3.18) | 165(52.55) | 131(41.7 | 1(0.24) | 9(2.19) | 20(4.87) | 211(51.34) | 170(41.3 | 1(0.31) | 6(1.87) | 6(1.87) | 168(52.34) | 140(43.61) | 0(0.00) | 11(1.57) | 26(3.71) | 358(51.0 | 306(43.65) | 0(0.00) | 2(4.76) | 2(4.76) | 27(64.29 | 11(26.19) |
| (10) How long the drug should be  taken | 0(0.00) | 5(2.44) | 21(10.24 | 101(49.27) | 78(38.05) | 1(0.32) | 11(3.50) | 20(6.37) | 168(53.50) | 114(36.3 | 0(0.00) | 12(2.92) | 23(5.60) | 226(54.99) | 150(36.5 | 2(0.62) | 1(0.31) | 11(3.43) | 169(52.65) | 138(42.99) | 1(0.14) | 17(2.43) | 28(3.99) | 390(55.6 | 265(37.80) | 0(0.00) | 1(2.38) | 1(2.38) | 25(59.52 | 15(35.71) |
| (11) How much dose the drug is  taken | 0(0.00) | 3(1.46) | 17(8.29) | 96(46.83) | 89(43.41) | 2(0.64) | 0(0.00) | 16(5.10) | 163(51.91) | 133(42.3 | 0(0.00) | 5(1.22) | 21(5.11) | 198(48.18) | 187(45.5 | 1(0.31) | 3(0.93) | 7(2.18) | 156(48.60) | 154(47.98) | 0(0.00) | 10(1.43) | 19(2.71) | 359(51.2 | 313(44.65) | 0(0.00) | 1(2.38) | 1(2.38) | 22(52.38 | 18(42.86) |
| **Information on adverse reactions** |  |  |  |  |  |  |  |  |  |  |  |  |  |  |  |  |  |  |  |  |  |  |  |  |  |  |  |  |  |  |
| (12) What are the side effects of the  drug | 2(0.98) | 14(6.83) | 27(13.17 | 97(47.32) | 65(31.71) | 1(0.32) | 8(2.55) | 34(10.83) | 169(53.82) | 102(32.4 | 2(0.49) | 17(4.14) | 38(9.25) | 203(49.39) | 151(36.7 | 2(0.62) | 8(2.49) | 19(5.92) | 166(51.71) | 126(39.25) | 1(0.14) | 19(2.71) | 52(7.42) | 369(52.6 | 260(37.09) | 0(0.00) | 1(2.38) | 3(7.14) | 23(54.76 | 15(35.71) |
| (13）What will cause the side effects of the drug | 2(0.98) | 18(8.78) | 40(19.51 | 87(42.44) | 58(28.29) | 4(1.27) | 21(6.69) | 43(13.69) | 150(47.77) | 96(30.57 | 2(0.49) | 29(7.06) | 56(13.63) | 191(46.47) | 133(32.3 | 2(0.62) | 10(3.12) | 38(11.84) | 156(48.60) | 115(35.83) | 1(0.14) | 33(4.71) | 97(13.84 | 341(48.6 | 229(32.67) | 0(0.00) | 3(7.14) | 2(4.76) | 26(61.90 | 11(26.19) |
| (14) What to do if you have side  effects | 0(0.00) | 12(5.85) | 33(16.10 | 91(44.39) | 69(33.66) | 2(0.64) | 15(4.78) | 38(12.10) | 156(49.68) | 103(32.8 | 1(0.24) | 25(6.08) | 47(11.44) | 190(46.23) | 148(36.0 | 2(0.62) | 11(3.43) | 49(15.26) | 142(44.24) | 117(36.45) | 1(0.14) | 24(3.42) | 89(12.70 | 333(47.5 | 254(36.23) | 0(0.00) | 0(0.00) | 3(7.14) | 19(45.24 | 20(47.62) |
| (15) What to do when you forgot to take the drug or when you took too  much | 1(0.49) | 17(8.29) | 39(19.02 | 91(44.39) | 57(27.80) | 5(1.59) | 25(7.96) | 58(18.47) | 138(43.95) | 88(28.03 | 4(0.97) | 39(9.49) | 64(15.57) | 174(42.34) | 130(31.6 | 4(1.25) | 18(5.61) | 44(13.71) | 152(47.35) | 103(32.09) | 2(0.29) | 40(5.71) | 138(19.6 | 318(45.3 | 203(28.96) | 0(0.00) | 1(2.38) | 9(21.43) | 19(45.24 | 13(30.95) |
| (16) What are the effects on your  kidney, heart, and life | 3(1.46) | 8(3.90) | 31(15.12 | 87(42.44) | 76(37.07) | 2(0.64) | 15(4.78) | 44(14.01) | 129(41.08) | 124(39.4 | 1(0.24) | 19(4.62) | 59(14.36) | 164(39.90) | 168(40.8 | 4(1.25) | 10(3.12) | 36(11.21) | 123(38.32) | 148(46.11) | 2(0.29) | 32(4.56) | 87(12.41 | 308(43.9 | 272(38.80) | 0(0.00) | 0(0.00) | 4(9.52) | 19(45.24 | 19(45.24) |
| (17) What are the allergies that the  drug can cause | 3(1.46) | 10(4.88) | 31(15.12 | 86(41.95) | 75(36.59) | 1(0.32) | 15(4.78) | 32(10.19) | 160(50.96) | 106(33.7 | 3(0.73) | 15(3.65) | 51(12.41) | 197(47.93) | 145(35.2 | 3(0.93) | 12(3.74) | 22(6.85) | 153(47.66) | 131(40.81) | 4(0.57) | 22(3.14) | 68(9.70) | 345(49.2 | 262(37.38) | 0(0.00) | 0(0.00) | 2(4.76) | 20(47.62 | 20(47.62) |
| **Effects on Daily Life** |  |  |  |  |  |  |  |  |  |  |  |  |  |  |  |  |  |  |  |  |  |  |  |  |  |  |  |  |  |  |
| (18) Whether the drug can make you  feel drowsy | 4(1.95) | 42(20.49) | 36(17.56 | 81(39.51) | 42(20.49) | 8(2.55) | 62(19.75) | 50(15.92) | 123(39.17) | 71(22.61 | 11(2.68) | 74(18.00 | 65(15.82) | 179(43.55) | 82(19.95 | 5(1.56) | 44(13.71) | 51(15.89) | 153(47.66) | 68(21.18) | 4(0.57) | 121(17.26 | 103(14.6 | 338(48.2 | 135(19.26) | 0(0.00) | 12(28.57 | 5(11.90) | 18(42.86 | 7(16.67) |
| (19) Whether you can drink alcohol  whilst taking the drug | 10(4.88) | 25(12.20) | 32(15.61 | 78(38.05) | 60(29.27) | 13(4.14) | 35(11.15) | 24(7.64) | 143(45.54) | 99(31.53 | 14(3.41) | 57(13.87 | 40(9.73) | 170(41.36) | 130(31.6 | 7(2.18) | 24(7.48) | 23(7.17) | 152(47.35) | 115(35.83) | 18(2.57) | 71(10.13) | 66(9.42) | 323(46.0 | 223(31.81) | 0(0.00) | 2(4.76) | 5(11.90) | 21(50.00 | 14(33.33) |
| (20) What are the impacts on driving  when taking the drug | 23(11.22) | 43(20.98) | 44(21.46 | 50(24.39) | 45(21.95) | 29(9.24) | 59(18.79) | 52(16.56) | 102(32.48) | 72(22.93 | 24(5.84) | 63(15.33 | 66(16.06) | 154(37.47) | 104(25.3 | 13(4.05) | 31(9.66) | 54(16.82) | 115(35.83) | 108(33.64) | 22(3.14) | 92(13.12) | 122(17.4 | 274(39.0 | 191(27.25) | 1(2.38) | 5(11.90) | 10(23.81) | 17(40.48 | 9(21.43) |
| (21) What are the impacts on your  reaction ability when taking the drug | 8(3.90) | 34(16.59) | 52(25.37 | 67(32.68) | 44(21.46) | 11(3.50) | 67(21.34) | 69(21.97) | 99(31.53) | 68(21.66 | 12(2.92) | 64(15.57 | 93(22.63) | 154(37.47) | 88(21.41 | 9(2.80) | 36(11.21) | 70(21.81) | 133(41.43) | 73(22.74) | 9(1.28) | 107(15.26 | 171(24.3 | 277(39.5 | 137(19.54) | 1(2.38) | 7(16.67) | 10(23.81) | 13(30.95 | 11(26.19) |
| (22) What are the impacts on your  diet when taking the drug | 2(0.98) | 8(3.90) | 35(17.07 | 100(48.78) | 60(29.27) | 6(1.91) | 51(16.24) | 46(14.65) | 142(45.22) | 69(21.97 | 6(1.46) | 40(9.73) | 61(14.84) | 209(50.85) | 95(23.11 | 5(1.56) | 31(9.66) | 34(10.59) | 168(52.34) | 83(25.86) | 5(0.71) | 88(12.55) | 102(14.5 | 349(49.7 | 157(22.40) | 0(0.00) | 3(7.14) | 6(14.29) | 25(59.52 | 8(19.05) |
| (23) Whether the drug will affect  your sex life | 27(13.17) | 46(22.44) | 68(33.17 | 38(18.54) | 26(12.68) | 40(12.74) | 89(28.34) | 87(27.71) | 64(20.38) | 34(10.83 | 44(10.71) | 111(27.0 | 123(29.93) | 81(19.71) | 52(12.65 | 21(6.54) | 72(22.43) | 92(28.66) | 88(27.41) | 48(14.95) | 64(9.13) | 160(22.82 | 227(32.3 | 164(23.4 | 86(12.27) | 3(7.14) | 5(11.90) | 13(30.95) | 11(26.19 | 10(23.81) |
| **Other information** |  |  |  |  |  |  |  |  |  |  |  |  |  |  |  |  |  |  |  |  |  |  |  |  |  |  |  |  |  |  |
| (24) How to get more drugs | 9(4.39) | 32(15.61) | 43(20.98 | 85(41.46) | 36(17.56) | 6(1.91) | 69(21.97) | 65(20.70) | 114(36.31) | 60(19.11 | 14(3.41) | 85(20.68 | 81(19.71) | 172(41.85) | 59(14.36 | 12(3.74) | 55(17.13) | 77(23.99) | 113(35.20) | 64(19.94) | 21(3.00) | 173(24.68 | 167(23.8 | 253(36.0 | 87(12.41) | 1(2.38) | 9(21.43) | 7(16.67) | 19(45.24 | 6(14.29) |
| (25) Whether the drug is reimbursed | 6(2.93) | 9(4.39) | 27(13.17 | 89(43.41) | 74(36.10) | 3(0.96) | 25(7.96) | 31(9.87) | 166(52.87) | 89(28.34 | 5(1.22) | 49(11.92 | 50(12.17) | 207(50.36) | 100(24.3 | 2(0.62) | 30(9.35) | 34(10.59) | 174(54.21) | 81(25.23) | 13(1.85) | 109(15.55 | 108(15.4 | 339(48.3 | 132(18.83) | 0(0.00) | 7(16.67) | 5(11.90) | 18(42.86 | 12(28.57) |
| (26) What are the reasons for different prescriptions when  suffering from same illness | 4(1.95) | 35(17.07) | 51(24.88 | 71(34.63) | 44(21.46) | 9(2.87) | 63(20.06) | 69(21.97) | 129(41.08) | 44(14.01 | 10(2.43) | 87(21.17 | 103(25.06) | 140(34.06) | 71(17.27 | 7(2.18) | 57(17.76) | 67(20.87) | 125(38.94) | 65(20.25) | 20(2.85) | 136(19.40 | 181(25.8 | 263(37.5 | 101(14.41) | 0(0.00) | 10(23.81 | 9(21.43) | 13(30.95 | 10(23.81) |
| (27) How to preserve the drug | 4(1.95) | 23(11.22) | 39(19.02 | 90(43.90) | 49(23.90) | 6(1.91) | 41(13.06) | 46(14.65) | 148(47.13) | 73(23.25 | 7(1.70) | 56(13.63 | 57(13.87) | 211(51.34) | 80(19.46 | 4(1.25) | 35(10.90) | 37(11.53) | 174(54.21) | 71(22.12) | 7(1.00) | 86(12.27) | 86(12.27 | 378(53.9 | 144(20.54) | 0(0.00) | 6(14.29) | 7(16.67) | 19(45.24 | 10(23.81) |
